# The lncRNA *MYRACL* regulates human oligodendrocyte maturation and myelination

**DOI:** 10.1016/j.ymthe.2025.08.011

**Published:** 2025-08-08

**Authors:** Themistoklis M. Tsarouchas, Francesca Vacante, Nina-Lydia Kazakou, Laura Wagstaff, Matthew Bennett, Lida Zoupi, Erin M. Gibson, Andrew H. Baker, Anna Williams

**Affiliations:** 1 Centre for Regenerative Medicine, Institute for Regeneration and Repair, University of Edinburgh, Edinburgh BioQuarter, EH16 4UU Edinburgh, UK; 2 Department of Psychiatry and Behavioral Sciences, Stanford University School of Medicine, Palo Alto, CA 94305, USA; 3 Queens Medical Research Institute, British Heart Foundation Centre for Research Excellence, Centre for Cardiovascular Sciences, University of Edinburgh, EH16 4TJ Edinburgh, UK; 4 Novo Nordisk Foundation for Stem Cell Medicine, reNEW, University of Copenhagen, Copenhagen 2200, Denmark; 5 Centre for Discovery Brain Sciences, The University of Edinburgh, EH8 9XD Edinburgh, UK; 6 Simons Initiative for the Developing Brain, University of Edinburgh, EH8 9XD Edinburgh, UK; 7 Department of Pathology, Cardiovascular Research Institute Maastricht School for Cardiovascular Diseases, Maastricht University, 6229 ER Maastricht, the Netherlands

**Keywords:** MYRACL, long non-coding RNA, lncRNA, oligodendrocyte differentiation, myelination, human embryonic stem cells, hESCs

## Abstract

Recent studies have described disease-associated expression patterns of long non-coding RNAs (lncRNAs) associated with neurodevelopment and neurodegeneration, highlighting their potential as regulators of function and therefore potential therapeutic targets. Oligodendrocyte (OL) dysfunction drives central nervous system myelin disruption in neurological disorders, but the mechanisms underlying impaired myelin patterns are still poorly understood. In this study, we uncover a role for the lncRNA MYRACL (myelination regulating oligodendrocyte-associated lncRNA) as a regulator of functional maturation and OL myelination. Analysis of RNA-sequencing data performed in human postmortem brain tissue revealed MYRACL to be among the top enriched genes expressed in the OL population compared to the OL precursor cell cluster. We validated this finding in an embryonic stem cell-derived oligodendroglia cell culture model. Analysis of evolutionary conservation and protein coding potential showed that MYRACL is non-coding and may exhibit conserved regions across mammalian species. Further co-expression analysis of lncRNAs-mRNAs suggested that expression of MYRACL positively correlates with genes known to be involved in driving oligodendroglia differentiation. GapmeR-mediated knockdown of nuclear MYRACL disrupted OL maturation *in vitro*, while lentivirus-mediated overexpression promoted OL differentiation with enhancement of myelin formation *in vitro*. Our findings highlight MYRACL as a novel regulatory mechanism in human OL maturation and myelination. By providing a human, translationally relevant platform, this work advances our ability to model human myelination *in vitro* and paves the way for precision medicine approaches targeting lncRNA-mediated dysregulation in neurodevelopmental and neurodegenerative diseases.

## Graphical abstract

**Figure:**
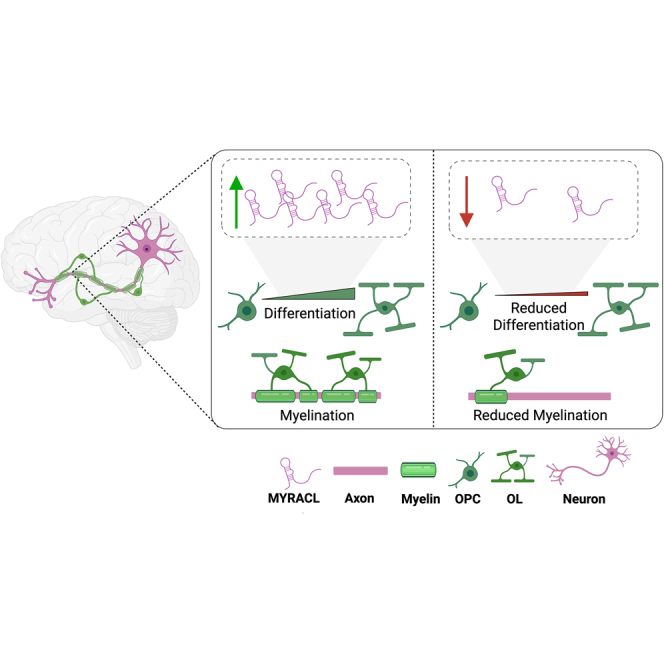

## Teaser

In this article, Tsarouchas, Vacante, and colleagues identify MYRACL, a brain-specific lncRNA that promotes human oligodendrocyte maturation and myelination *in vitro* and *ex vivo*, highlighting it as a novel regulator of myelin formation and a potential therapeutic target for remyelination in demyelinating diseases.

## Introduction

Oligodendrocyte (OL) differentiation is crucial for central nervous system (CNS) function, particularly in the formation of myelin, which enables rapid and efficient electrical signal conduction along axons.^1^ This complex process involves a tightly regulated interplay of signaling pathways, growth factors, and transcription factors that collectively guide the maturation of OL precursor cells (OPCs) into myelinating OLs.^2^ A two-tier transcriptional control mechanism has been described, wherein epigenetic repression of inhibitory genes coincides with activation of myelin-related gene expression to drive differentiation.^3^ Given that OPCs persist into adulthood and can respond to injury or disease, disruptions in their differentiation are increasingly being recognized as contributing factors to neurodegenerative and psychiatric disorders.^1^ Amid growing interest in the regulatory complexity of CNS development, long non-coding RNAs (lncRNAs) have emerged as important modulators of gene expression.^4^ Approximately 40% of all lncRNAs are enriched in the brain.^5^^,^^6^ Examples include *OLMALINC* and lnc-*PINT*, two conserved nuclear lncRNAs involved in OL maturation and the regulation of cell proliferation, oxidative stress, and apoptosis, respectively.^7^^,^^8^ Additionally, the lncRNAs *Gomafu* and *Neat1* have been shown to be expressed throughout the oligodendroglia lineage progression and appear to be upregulated during OL lineage specification and maturation.^9^ Similarly, lncRNAs *PNKY* and *PAUPAR* play critical roles in neurogenesis, shaping transcriptional networks and chromatin dynamics to guide neural stem cell differentiation and cortical development.^5^^,^^10^ lncRNAs have also been implicated in neurological disorders characterized by aberrant myelination. For example, *BACE1-AS*, a circulating biomarker in Alzheimer disease (AD), stabilizes *BACE1* mRNA leading to enhanced Aβ amyloid deposition in AD.^11^ Additionally, lncRNAs are known for regulating microRNAs in health and disease.^12^ Examples include *KCNQ1OT1* and *SNHG1*, which control NLRP3 inflammasomes via the regulation of miR-30e-3p and miR-7, respectively, in multiple sclerosis (MS)^13^ and Parkinson disease (PD).^14^ In murine OLs, lnc*OL1* interacts with SUZ12 and regulates myelination in the developing brain.^15^ Additionally, lnc-*OPC* has been found to be regulated by OLIG2^16^ in the mouse brain, and this interaction is essential for OPC generation, whereas the lncRNA *TubAR* has been shown to interact with TUBB4A and TUBA1A to facilitate microtubule assembly and support of the maintenance of myelination.^17^ This evidence highlights the value of lncRNAs as potential therapeutic targets for neurological disorders.

Despite these insights, the role of lncRNAs in human oligodendroglia development, differentiation, and myelination remains largely unknown, primarily due to the decreased conservation between mouse and human lncRNAs^18^ and the limited functional human data and reliable human models that can accurately recapitulate human oligodendroglial biology. Human embryonic stem cells (hESCs) are a powerful tool for the study of development and maturation processes as well as pro-pathological phenotypes, providing a valuable platform to effectively assess mechanisms controlling cell physiology and disease.^19^ In this study, using *in vitro* cultures of ESC-derived OPCs and OLs combined with *ex vivo* mouse cultures, we identified *MYRACL* (myelination regulating oligodendrocyte-associated lncRNA), a nuclear brain-specific long intergenic RNA (lincRNA), as a regulator of human OL maturation and myelination.

## Results

### *MYRACL* is a CNS-specific lncRNA enriched during the differentiation of human OLs

**Figure 1:**
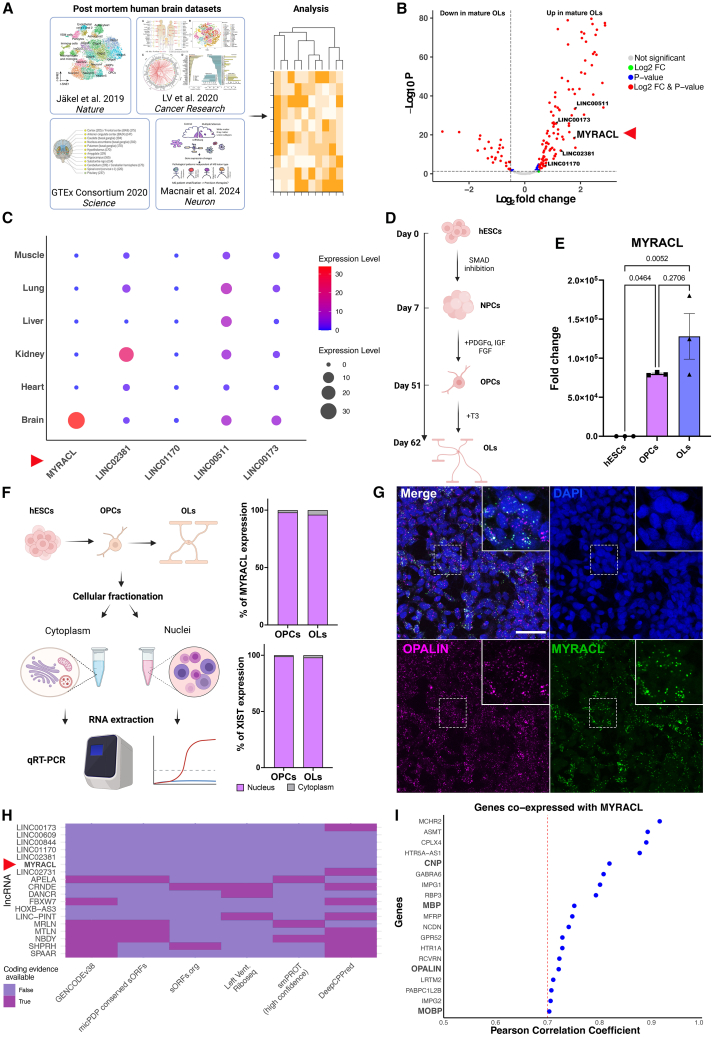
*MYRACL* is a brain-specific nuclear lncRNA upregulated in mature oligodendrocytes (A) Schematic representation of the pipeline used to re-analyze three published snRNA-seq datasets from postmortem brain tissue and correlated with the LncSpA atlas. (B) Volcano plot displaying the top differentially expressed lncRNAs in mature oligodendrocytes (OLs) versus OPC stage. (C) Bubble plot showing tissue-specific expression profiles for the top enriched lncRNAs identified. (D) Schematic of hESC-derived OPC and OL differentiation protocol (62 days). (E) RT-qPCR analysis showing increased *MYRACL* expression moving from hESCs to OPCs to OLs (*n* = 3), one-way ANOVA (F(2,6) = 8.946) followed by Bonferroni multiple comparisons. (F) Schematic and RT-qPCR data of *MYRACL* expression showing more nuclear location compared to cytoplasmic regions following cellular fractionation of hESC-derived OPCs and OLs. *XIST*, a well-characterized nuclear lncRNA, served as a positive control for nuclear enrichment (*n* = 1). (G) RNA-Scope *in situ* hybridization on hESC-derived oligodendroglia. *MYRACL* is expressed by *OPALIN*^+^ OLs. Dashed rectangles indicate a representative area within the culture that is visualized in higher magnification on the top right of the panel. (H and I) Scale bar, 50 μm. (H) Evidence of the non-coding status of *MYRACL* and other OL-enriched lncRNAs in selected databases relevant for coding potential and/or micropeptide production alongside positive controls of previously annotated lncRNAs with strong evidence of micropeptide production. (I) Co-expression analysis of *MYRACL* showing positive correlation with OL markers *MBP, MOBP,OPALIN,* and *CNP* enriched in OPC to OL differentiation (Pearson correlation >0.7).

To identify lncRNAs whose expression pattern is altered during oligodendroglia differentiation, we re-analyzed publicly available relevant single-nucleus RNA sequencing (snRNA-seq) and spatial transcriptomics datasets from human postmortem brain tissues, encompassing transitions from OPCs to mature OLs (Figure 1A). Using non-coding annotations present in the reference transcriptome, this analysis identified *MYRACL,* *LINC00173,* *LINC02381,* *LINC00511*, and *LINC01170* among the top enriched lncRNAs during this transition (Figure 1B; Table S2). As lncRNAs frequently exhibit tissue-specific expression patterns, we leveraged LncSpA, a tissue-specific atlas of human lncRNAs.^20^ This pinpointed *MYRACL* as a highly expressed lncRNA in brain tissue compared to other identified lncRNAs (Figure 1C). *MYRACL* is a lincRNA, currently annotated as *LINC02488* (ENSEMBL: ENSG00000249362), located in position chr5:87,607,623–87,933,811 of the human genome (GENCODE version 47). By looking at the syntenic region of the mouse genome, we have also identified two regions proximal to the transcriptional start site with elevated phyloP scoring (mean of 1.36 for mm10:chr13:84,931,280–84,931,648 and mean of 1.85 for mm10:chr13:84,933,017–84,933,225), suggesting potential conservation across placental mammals. To further validate the *MYRACL* expression profile, we used an established *in vitro* differentiation platform in which hESCs are directed to differentiate into OPCs and subsequently into OLs within a period of 62 days (Figure 1D). Quantitative reverse transcription PCR (RT-qPCR) analysis showed a progressive increase in *MYRACL* expression during the differentiation process (Figure 1E). We validated the expression patterns of all identified lncRNAs during *in vitro* differentiation and observed that only *MYRACL* exhibited consistent upregulation during the transition from OPCs to OLs (data not shown). These results directed our focus on the study of *MYRACL* as a brain-specific lncRNA. To gain insights into the cellular localization of *MYRACL*, which can provide information on its potential cellular function, we performed fractionation of human oligodendroglia and found that *MYRACL* predominantly localized in the nucleus of both hESC-derived OPCs and OLs (Figure 1F). We used *XIST*, a well-characterized nuclear lncRNA, as a positive control to confirm accuracy of fractionation (Figure 1F). To gain information on the spatial localization of *MYRACL*, we performed RNA-Scope *in situ* hybridization on hESC-derived oligodendroglia. *MYRACL* showed robust expression in mature OLs, as evidenced by its co-localization with the OL marker *OPALIN*, with cytoplasmic localization (Figure 1G). As lncRNAs are often re-annotated as coding genes after uncovering evidence of micropeptide production,^21^ we checked this possibility for *MYRACL* using available resources that assess this possibility.^22^^,^^23^ *MYRACL* and other OL-enriched lncRNAs still lacked any evidence of coding potential compared to genes with strong evidence of micropeptide production, such as *SPAAR*,^24^ *DANCR*,^25^ and *APELA*^26^ (Figures 1H; Table S4). Correlation analysis of *MYRACL* expression with other known regulators of oligodendroglia dynamics enriched in OPC to OL differentiation, combined with the information from the LncSPA atlas, showed a positive correlation between *MYRACL* and OL markers, within the three datasets, including *MBP,* *OPALIN, MOBP*, and *CNP* (Figures 1I; Table S3).

### Depletion of *MYRACL* impairs human OPC to OL differentiation *in vitro*

**Figure 2:**
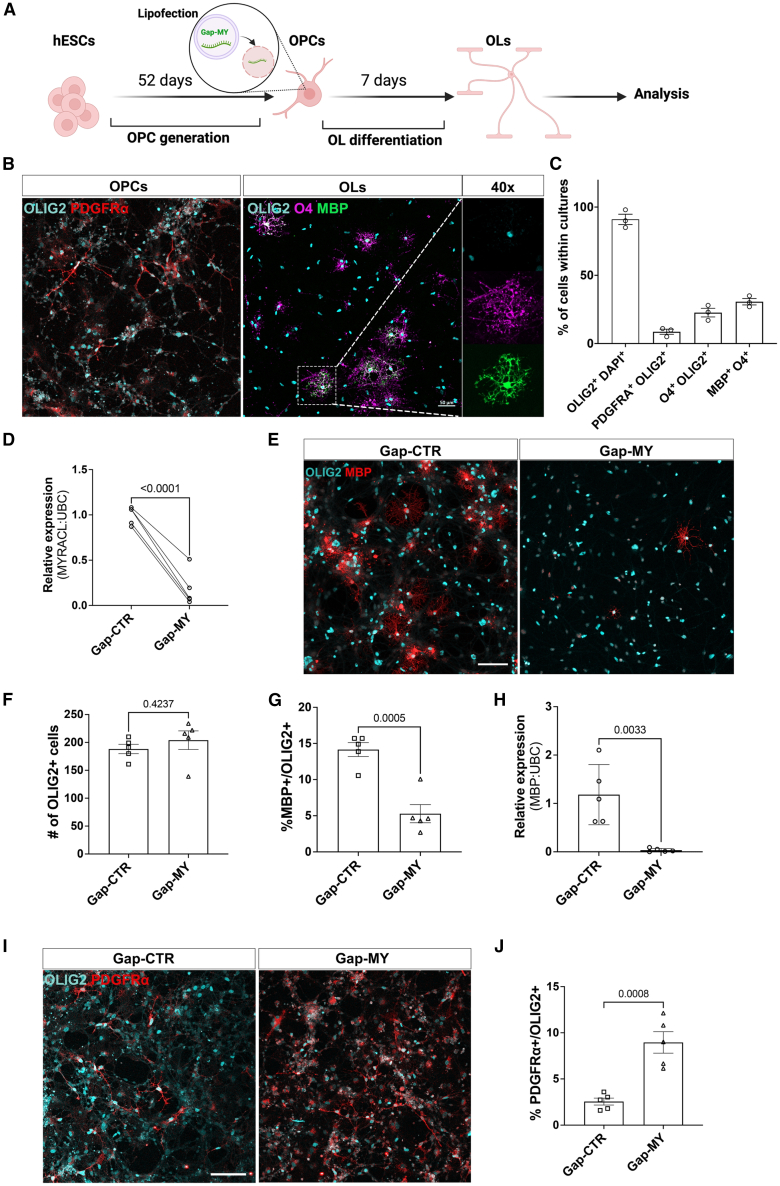
GapmeR-mediated *MYRACL* knockdown decreases MBP expression while increasing PDGFRα-expressing cells (A) Schematic of *in vitro* depletion of *MYRACL* during the hESC-OPC to OL differentiation process. (B) Example micrographs of the generated OPCs and OLs generated *in vitro*. The dashed rectangle indicates a selected OL that expresses OLIG2, O4, and MBP and is visualized in higher magnification on the right side of the panel. (C) Quantification of *in vitro* differentiation efficiency (*n* = 3). (D) RT-qPCR of *MYRACL* following Gap-MY treatment of OPC versus Gap-CTR following 7 days of treatment (*n* = 5), unpaired two-tailed t test (t = 8.285, df = 8). (E) Representative photomicrographs of OLIG2^+^/MBP^+^ oligodendroglial cells after Gap-MY treatment or control (scale bar, 20 μm) (*n* = 5). (F and G) Quantification of IF staining of OLIG2^+^ cells, unpaired two-tailed t test (t = 0.8431, df = 8), mature OLs (OLIG2^+^/MBP^+^ cells) (*n* = 5), and unpaired two-tailed t test (t = 5.624, df = 8). (H) RT-qPCR of *MBP* following treatment with Gap-MY or control (*n* = 5), unpaired two-tailed t test (t = 3.574, df = 8). (I and J) Micrographs of OLIG2^+^/PDGFRα^+^ in *MYRACL*-depleted OLs or control and quantification of cell densities (*n* = 5), unpaired two-tailed t test (t = 5.261, df = 8). All Scale bars, 50 μm. Bars show mean with SEM for all and *p* values given on graphs.

To assess the role of *MYRACL* in human oligodendroglia differentiation and myelination, we performed depletion of its expression using GapmeR technology in OPC to OL differentiation (Figures 2A–2C). We first evaluated the GapmeR-mediated knockdown efficiency and confirmed significant downregulation of *MYRACL* following transfection with *MYRACL* GapmeR (Gap-MY) compared to control (Gap-CTR) (Figure 2D). We then assessed the functional consequence of *MYRACL* depletion by assessing oligodendroglia generation and OL differentiation through immunofluorescence (IF) staining and RT-qPCR for OLIG2 and myelin basic protein (*MBP*), respectively. While the number of OLIG2^+^ cells was not affected following treatment with Gap-MY, indicating no effects on the generation of oligodendroglial lineage cells, the depletion of *MYRACL* led to decreased levels of *MBP* at the transcriptional and MBP^+^ cell density levels (Figures 2E–2H). Additionally, *MYRACL*-depleted cells exhibited a significant increase in the proportion of platelet-derived growth factor receptor α^+^ (PDGFRα^+^)/OLIG2^+^ cells (Figures 2I and 2J). As PDGFRα is a marker of the OPC stage, these results suggest a decrease in the maturation progression toward mature OLs.

### Overexpression of *MYRACL* regulates human OL differentiation and myelination *in vitro* and *ex vivo*

**Figure 3:**
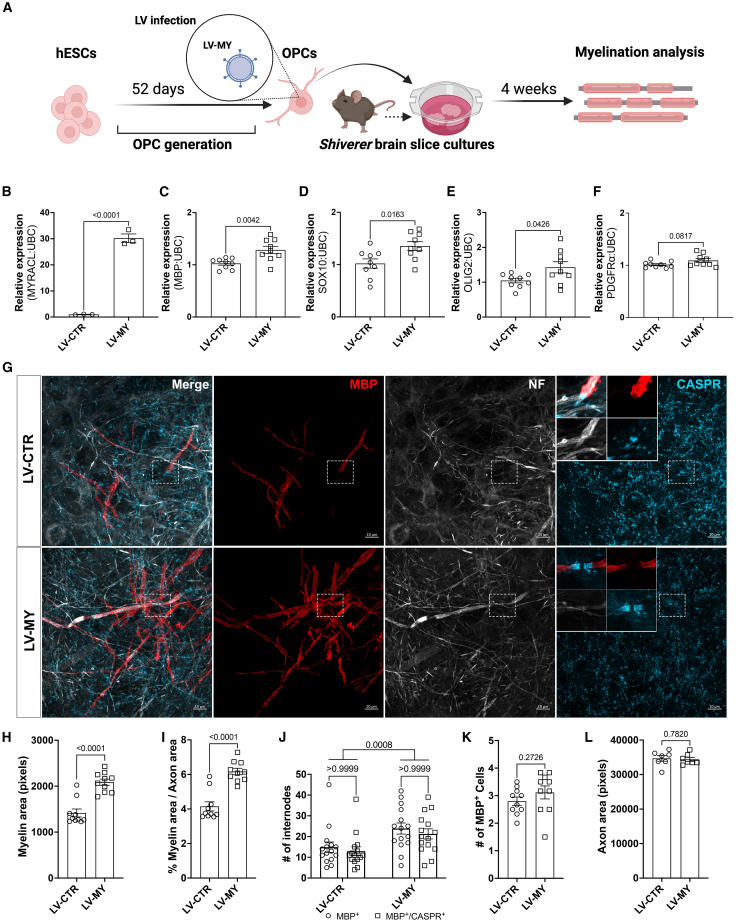
Lentiviral-mediated overexpression of *MYRACL* enhances human OL differentiation and myelination (A) The experimental setup of lentiviral transduction of OPC cultures and subsequent transplantation onto *Shiverer* brain slice cultures for myelination assessment. (B–F) RT-qPCR of *MYRACL*, unpaired two-tailed t test (t = 15.11, df = 4); *MBP*, unpaired two-tailed t test (t = 3.243, df = 16); *SOX10*, unpaired two-tailed t test (t = 2.887, df = 16); *OLIG2*, unpaired two-tailed t test (t = 2.182, df = 16); and *PDGFRA*, unpaired two-tailed t test (t = 1.843, df = 16) (*n* = 9) after OPC transduction with LV-MY versus control (LV-CTR). (G) Representative photomicrographs of IF for neurofilament (NF), MBP, and CASPR in hESC-derived OLs after *MYRACL* overexpression. Maximum intensity projections visualize the increased number of MBP^+^ myelin segments after *MYRACL* overexpression. Dashed rectangles indicate a selected region, which is presented in higher magnification as single optical section on the top left of the CASPR panel, visualizing the CASPR localization. (H and I) Quantification of total myelin area; unpaired two-tailed t test (t = 6.107, df = 18) and myelin relative to axonal density; unpaired two-tailed t test (t = 6.204, df = 18) following lentiviral treatment (26 sections from *n* = 10 animals). (J) Quantification of MBP^+^ internodes with paranodal loops (CASPR^+^) after *MYRACL* lentiviral overexpression. No significant difference is observed between the numbers of MBP^+^/CASPR^+^ and MBP^+^ internodes within the LV-CTR and the LV-MY groups; however, the total number of internodes is increased after the lentiviral overexpression of *MYRACL*; two-way ANOVA (F(1,56) = 12.57, *p* = 0.0008). (K and L) Quantification of MBP^+^ cells; unpaired two-tailed t test (t = 1.132, df = 18) and total axonal area; unpaired two-tailed t test (t = 0.2830, df = 12) following lentiviral transductions (26 sections from *n* = 10 animals). Scale bar, 10 μm. All bars show means ± SEM and *p* values given on graphs.

Given that depletion of *MYRACL* decreases the differentiation of human OLs, we next sought to understand the functional consequences of *MYRACL* overexpression. To efficiently enhance its expression, we generated a lentiviral vector (LV) carrying the lncRNA transcript (*GRCh38.p13*) named LV-MY. hESC-derived OPCs were transduced with either LV-MY or an empty control lentivirus (LV-CTR) for 48 h (Figure 3A). Lentiviral transduction efficiency was then assessed by RT-qPCR analysis, confirming overexpression of the lncRNA compared to the control lentivirus (Figure 3B). To determine the functional consequence of *MYRACL* overexpression on OL maturation, we examined the expression of key OL lineage markers, including *SOX10*, *OLIG2*, *PDGFRα*, and *MBP*, by RT-qPCR analysis. Lentiviral-mediated overexpression resulted in a significant upregulation of *SOX10* and *OLIG2*, two critical transcription factors required for OL differentiation, along with an increase in *MBP* expression, indicating enhanced progression to the myelinating stage. In contrast, LV-02488 did not alter the expression of the OPC marker PDGFRα compared to control (Figures 3C–3F). To assess the effects of *MYRACL* overexpression on myelination, transduced OPCs were transplanted onto organotypic brain slice cultures derived from *Shiverer* *(MBP*^*shi/shi*^) mice, a model that lacks functional MBP and thus lacks compact myelin due to a mutation in the *MBP* gene (Figure 3A). We leveraged the *Shiverer* brain slices *ex vivo* culture model^27^ because MBP^+^ myelin formed in these cultures is by definition derived from hESC-derived OPCs and because the three-dimensional structure is a more physiologically relevant environment. At 4 weeks post-transplantation, overexpression of *MYRACL* enhanced MBP protein expression and myelin sheath formation, as shown by the quantification of MBP coverage normalized to the axonal area. Colocalization of MBP and contactin-associated protein (CASPR)^28^ at the myelin segments indicates the generation of myelin sheaths after the overexpression of *MYRACL* (Figures 3G–3J). However, the total number of MBP^+^ cells was unaffected, indicating that the overexpression of *MYRACL* altered the amount of myelin sheaths rather than OL number (Figure 3K). This was not dependent on axon availability, which remained unchanged across conditions (Figure 3L).

Therefore, the above data from gain- and loss-of-function experiments in human oligodendroglia indicate that *MYRACL* is important for differentiation and myelin sheath formation.

## Discussion

Given their broad regulatory roles in cellular processes, lncRNAs are increasingly recognized as key modulators of oligodendroglial dynamics and differentiation within the CNS.^15^^,^^16^^,^^17^^,^^29^ In this study, we identified *MYRACL* as a novel candicate lncRNA that plays an important role in the maturation of human OLs and the process of myelination *in vitro*. In addition to OLs, *MYRACL* shows detectable expression in inhibitory neurons, while its expression is absent in excitatory neurons, astrocytes, and microglia. Our data provide insights into *MYRACL* expression profiles throughout human oligodendroglia differentiation, tissue-specific spatial expression in human biospecimens, coding potential, and subcellular localization. We show that *MYRACL* expression increases during the differentiation of human OLs, suggesting its involvement in the maturation process. We were able to effectively modulate the expression of *MYRACL* in gain- and loss-of-function studies, showing that *MYRACL* overexpression promoted OL differentiation and enhanced myelin formation *in vitro*, whereas depletion impaired OL differentiation.

The nuclear localization of *MYRACL* suggests possible roles associated with epigenetic or post-transcriptional regulation of gene expression.^4^^,^^5^^,^^6^ Given the established role of lncRNAs in tuning gene expression during neural development,^5^ we conducted a co-expression analysis to explore the potential pathways in which *MYRACL* may be functionally involved. The results showed a positive correlation between *MYRACL* and the canonical markers of mature OLs *MBP, MOBP, CNP,* and *OPALIN*. While this might suggest potential involvement in similar pathways, further validation studies are needed to confirm the functional relevance of these associations. Additionally, we found that overexpressing *MYRACL* led to increased expression of MBP, SOX10, and OLIG2 while PDGFRα levels remained unchanged. *MBP* expression was also positively correlated with *MYRACL* levels (Figure 1I), but no such correlation was seen for *SOX10,* *OLIG2*, or *PDGFRA*. These results suggest that the lncRNA may support the transition toward a more mature OL state, rather than affecting early lineage specification. It is not yet known whether a murine *MYRACL* homolog exists with a similar function or whether this is a species-specific regulatory pathway.

Understanding the mechanisms by which *MYRACL* controls OL maturation could provide new insights into therapeutic strategies for neurological disorders characterized by aberrant myelination in development such as in autism and by demyelination (needing remyelination) such as in MS and AD. We identified *MYRACL* from RNA-seq datasets of control and MS donors, but whether *MYRACL* expression is altered in other diseases with myelin deficit is unknown and could provide additional insights into to whether lncRNAs could act as a general target for promoting (re)myelination and restoring neural function in disease.^4^^,^^7^^,^^13^^,^^15^^,^^17^ Additionally, overexpression of *MYRACL* to improve the efficiency of human *in vitro* differentiation methods may be useful in assays for regenerative medicine research.

Although lentiviral overexpression of genes is efficient for functional research, its genomic integration presents challenges for therapeutic applications in humans. Alternate approaches, including non-integrating systems such as adeno-associated virus or lipid nanoparticles, or CRISPR-based endogenous genomic regulatory element activation, could provide more clinically relevant means of altering *MYRACL* expression. Mechanistic studies are required to dissect the specific role of *MYRACL* within the oligodendroglial transcriptional network, particularly in relation to key myelin-associated genes such as *MBP, MOBP,* *OPALIN*, and *CNP*, which may act either as targets, co-regulated partners, or downstream effectors. Understanding how *MYRACL* interfaces with these canonical markers will be essential for delineating its contribution to the molecular architecture of myelination.

In conclusion, we identified *MYRACL* as a novel regulator of human OL maturation and myelination, which may enhance our understanding of human myelin disorders and pave the way for targeted therapeutic strategies.

## Materials and methods

This information can be found in the supplemental information.

## Data availability

Data are included within the paper. Raw data collected are available from the corresponding authors upon request.

## Acknowledgments

The authors wish to thank Dr. Pamela Brown (IRR Biomolecular Core, University of Edinburgh) for her technical assistance generating the viral vectors used in this study. This study was supported by the Multiple Sclerosis (MS) Society UK (A.W.), the Medical Research Council (MRC) (MR/P016022/1) (A.W.), the MRC-MS Society UK (MR/T015594/1) (A.W.), the British Heart Foundation (BHF) (ReGenLnc) (A.H.B.), British Heart Foundation Programme grant no. RG/20/5/34796 (A.H.B.), and the BHF Chair of Translational Cardiovascular Sciences, Wellcome Trust iTPA PIII-020SF (T.M.T. and F.V.). For the purpose of open access, the author has applied a Creative Commons Attribution (CC BY) license to this manuscript.

## Author contributions

Conceptualization and methodology, T.M.T., F.V., A.H.B., and A.W. Investigation and analysis, T.M.T., F.V., N.-L.K., L.W., M.B., and L.Z. Writing – original draft, T.M.T. F.V., A.H.B., and A.W. Writing – review & editing, T.M.T., F.V., N.-L.K., L.W., M.B., L.Z., E.M.G., A.H.B., and A.W. Visualization, T.M.T. and F.V. Funding acquisition, T.M.T., F.V., A.H.B., and A.W. Supervision, A.H.B. and A.W.

## Declaration of interests

The authors declare no competing interests.

## Supplemental information

Document S1. Tables S1, S3, and S4 and supplemental methods

Table S2. List of differentially expressed lncRNAs across datasets

Document S2. Article plus supplemental information
