# Supplementary material for: The lncRNA MYRACL regulates human oligodendrocyte maturation and myelination
Source: Mol Ther. 2025 Aug 8;33(12):6025–32. doi: 10.1016/j.ymthe.2025.08.011 (PMC12703153; doi:10.1016/j.ymthe.2025.08.011)
Supplement: Document S1. Tables S1, S3, and S4 and supplemental methods [file mmc1.pdf]

YMTHE, Volume *33*

Supplemental Information

The lncRNA *MYRACL* regulates human oligodendrocyte maturation and myelination

Themistoklis M. Tsarouchas, Francesca Vacante, Nina-Lydia Kazakou, Laura Wagstaff, Matthew Bennett, Lida Zoupi, Erin M. Gibson, Andrew H. Baker, and Anna Williams

Supplemental Tables

Table S1. qRT-PCR primer efficiency calculated using the "(E = (10^(-1/slope)) – 1) formula.

Primer	Slope	% Efficiency
UBC	-3.282	101.7
MYRACL	-3.2111	104.8
MBP	-3.1677	106.9
SOX10	-3.0131	114.7
OLIG2	-3.0886	110.8
PDGFRα	-3.1583	107.3

Table S2. List of differentially expressed lncRNAs across datasets. (Can be found in the attached file).

Table S3. Correlation analysis between MYRACL and protein-coding genes in human single-nucleus RNA-seq data. The table displays the top positively and negatively correlated protein-coding genes, along with their Ensembl gene IDs, gene symbols, correlation coefficients, and associated p-values.

Ensembl_ID lncRNA	lncRNA_Symbol	Ensembl_ID mRNA Gene	mRNA_Gene_Symbol	Pearson_correlation	Pval
ENSG00000249362	MYRACL	ENSG00000152034	MCHR2	0.9252	0
ENSG00000249362	MYRACL	ENSG00000292336	ASMT	0.8959	0
ENSG00000249362	MYRACL	ENSG00000166569	CPLX4	0.8823	0
ENSG00000249362	MYRACL	ENSG00000220575	HTR5A-AS1	0.8662	0
ENSG00000249362	MYRACL	ENSG00000173786	CNP	0.8201	0
ENSG00000249362	MYRACL	ENSG00000145863	GABRA6	0.8108	0

ENSG00000249362	MYRACL	ENSG00000112706	IMPG1	0.8022	0
ENSG00000249362	MYRACL	ENSG00000265203	RBP3	0.7979	0
ENSG00000249362	MYRACL	ENSG00000197971	MBP	0.7524	0
ENSG00000249362	MYRACL	ENSG00000235718	MFRP	0.7445	0
ENSG00000249362	MYRACL	ENSG00000020129	NCDN	0.7417	0
ENSG00000249362	MYRACL	ENSG00000178394	HTR1A	0.7381	0
ENSG00000249362	MYRACL	ENSG00000203737	GPR52	0.7335	0
ENSG00000249362	MYRACL	ENSG00000109047	RCVRN	0.7212	0
ENSG00000249362	MYRACL	ENSG00000197430	OPALIN	0.7154	0
ENSG00000249362	MYRACL	ENSG00000166159	LRTM2	0.7100	0
ENSG00000249362	MYRACL	ENSG00000184388	PABPC1L2B	0.7092	0
ENSG00000249362	MYRACL	ENSG00000006116	CACNG3	0.7086	0
ENSG00000249362	MYRACL	ENSG00000081148	IMPG2	0.7058	0
ENSG00000249362	MYRACL	ENSG00000168314	MOBP	0.7044	0
ENSG00000249362	MYRACL	ENSG00000125835	SNRPB	-0.9507	8.41E-17
ENSG00000249362	MYRACL	ENSG00000145721	LIX1	-0.9511	7.56E-17
ENSG00000249362	MYRACL	ENSG00000262655	SPON1	-0.9512	7.17E-17
ENSG00000249362	MYRACL	ENSG00000164611	PTTG1	-0.9530	4.17E-17
ENSG00000249362	MYRACL	ENSG00000125170	DOK4	-0.9537	3.38E-17
ENSG00000249362	MYRACL	ENSG00000114698	PLSCR4	-0.9549	2.28E-17
ENSG00000249362	MYRACL	ENSG00000115457	IGFBP2	-0.9560	1.57E-17
ENSG00000249362	MYRACL	ENSG00000125354	SEPTIN6	-0.9562	1.47E-17
ENSG00000249362	MYRACL	ENSG00000165802	NSMF	-0.9565	1.33E-17
ENSG00000249362	MYRACL	ENSG00000127084	FGD3	-0.9616	2.17E-18

Table S4. Summary of coding potential analysis for all transcript isoforms of the lncRNA MYRACL using the CPC2 tool. Each transcript is annotated with its Ensembl Transcript ID, predicted peptide length (if any), Fickett score (reflecting positional nucleotide bias), isoelectric point (pI), ORF integrity (1 = intact ORF, -1 = lacking intact ORF), and predicted coding probability. All transcript isoforms are labeled as noncoding by CPC2, consistent with GENCODE annotations.

Transcript_ID	Peptide length	Fickett_ score	pI	ORF integrity	Coding probability	Label
ENST00000504287.3	57	0.30883	9.60064697	1	0.0258124	noncoding
ENST00000655396.2	82	0.24797	9.6317749	1	0.0585032	noncoding
ENST00000815382.1	20	0.36105	10.3098755	-1	0.134737	noncoding
ENST00000815383.1	19	0.38708	10.3098755	-1	0.10864	noncoding
ENST00000815384.1	20	0.34969	10.3098755	-1	0.147547	noncoding
ENST00000815385.1	20	0.37967	10.3098755	-1	0.115735	noncoding
ENST00000815386.1	20	0.38394	10.3098755	-1	0.111859	noncoding
ENST00000815387.1	20	0.33139	10.3098755	-1	0.168443	noncoding
ENST00000815388.1	20	0.4197	10.3098755	-1	0.0874846	noncoding
ENST00000815389.1	20	0.35712	10.3098755	-1	0.139098	noncoding
ENST00000815390.1	20	0.40561	10.3098755	-1	0.0953708	noncoding
ENST00000815391.1	19	0.33386	10.3098755	-1	0.165638	noncoding
ENST00000815392.1	19	0.41544	8.34283447	1	0.00552503	noncoding
ENST00000815393.1	14	0.45961	10.8344116	1	0.0181856	noncoding
ENST00000815394.1	82	0.2613	9.6317749	1	0.0534975	noncoding
ENST00000815395.1	40	0.25242	5.59637451	1	0.0359882	noncoding
ENST00000815396.1	40	0.27616	5.59637451	1	0.0196133	noncoding
ENST00000815397.1	40	0.30862	5.59637451	1	0.012922	noncoding

ENST00000815398.1	38	0.29826	9.90155029	1	0.0153645	noncoding
ENST00000815399.1	45	0.30763	6.03131104	1	0.0144505	noncoding

Supplemental Methods

Generation of OPCs and Oligodendrocytes from hESCs in-vitro

We obtained ethical permission from the UK Stem Cell Bank for the use of human embryonic stem cells (hESCs). These cells have normal karyotype and have recently undergone single nucleotide polymorphism analysis (SNP) analysis as previously described [1]. Generation of hESC-derived OPCs and OLs was performed as previously described [2]. hESCs were cultured on laminin-521-coated plates (5 µg/ml, Biolamina) using StemMACS™ iPS-Brew XF medium (Miltenyi Biotec) supplemented with 1% Antibiotic Antimycotic Solution (Sigma). To initiate differentiation, hESCs were detached using accutase and resuspended in StemMACS™ iPS-Brew XF medium with 1% Antibiotic Antimycotic Solution and ROCK inhibitor Y-27632 (10 µM, Tocris). Approximately 2 × 10^6 cells were seeded per well in AggreWell™400 microwell culture plates (Stem Cell Technologies) and incubated overnight to form embryoid bodies. Next, the embryoid bodies were transferred to a rotary shaker, and cultured in a chemically defined neuralization medium containing a 1:1 mix of F12 and Iscove's modified Dulbecco's medium (Invitrogen), with chemically defined Lipid concentrate 100 (Invitrogen), BSA (5 mg/ml, Sigma), monothioglycerol (450 µM, Sigma), insulin (7 mg/ml, Roche), transferrin (15 mg/ml, Roche), and 1% Antibiotic Antimycotic Solution. Activin inhibitor SB 431542 (10 µM, Sigma), N-acetyl cysteine (1 mM, Sigma), and LDN193189 (0.1 µM, Stratech) were added for neural induction. After 10 days, the neural spheres were caudalized for an additional 7 days in chemically defined medium

supplemented with N-acetyl cysteine (1 mM, Sigma), heparin (5 μg/ml, Sigma), retinoic acid (0.1 μM, Sigma), and basic fibroblast growth factor (FGF-2; 10 ng/ml, PeproTech). Neural conversion was assessed morphologically when cells were plated on laminin-coated plates (10 μg/ml, Sigma). Following neural induction, the spheres were cultured in advanced DMEM medium containing 0.5% GlutaMAX (Invitrogen), 1% N2 (Invitrogen), 1% B27™ (Invitrogen), 5 μg/ml heparin (Sigma), and 1% Antibiotic Antimycotic Solution. For ventralization, the medium was supplemented with purmorphamine (1 μM, Calbiochem), retinoic acid (1 μM, Sigma), and FGF-2 (10 ng/ml, PeproTech) for 7 days, after which FGF-2 was withdrawn for 14 days. Next, hOPC proliferation was enhanced by adding FGF-2 (10 ng/ml), alongside T3 (60 ng/ml, Sigma), PDGFα (20 ng/ml, PeproTech), SAG (1 μM, Calbiochem), purmorphamine (1 μM, Sigma), and IGF-1 (10 ng/ml, PeproTech). After 2 weeks, the spheres were dissociated using the Worthington papain dissociation system according to the manufacturer's protocol. Cells were plated at a density of 4×10^4 cells in 40 μl droplets on coverslips coated with poly-ornithine (1:100, Sigma), laminin (10 μg/ml, Sigma), fibronectin (20 μg/ml, Sigma), and Matrigel (Corning), whereas 1×10^6 cells per well were used for coated 6-well plates. For final differentiation into oligodendrocytes, cells were maintained for 1 week in advanced DMEM supplemented with 0.5% GlutaMAX, 1% N2, 1% B27, heparin (5 μg/ml), and 1% Antibiotic Antimycotic Solution, along with IGF-1 (10 μg/ml, PeproTech), T3 (60 μg/ml, Sigma), and ITS (Insulin-Transferrin-Sodium Selenite; 1:100, Sigma).

Immunofluorescence

Coverslips were rinsed with PBS and fixed with 4% PFA for 10 minutes. After fixation, coverslips were washed with PBS and blocked in a solution of PBS containing 10% horse serum (v/v) and 0.1% Triton-X (v/v) for 1 hour at room temperature. Primary antibodies anti-MBP (rat monoclonal, MCA409S, BioRad, 1:250), anti-Olig2 (goat polyclonal, AF2418, R&D systems, 1:400), anti-O4 (mouse monoclonal, MAB1326, R&D systems, 1:1000), anti-PDGFRα (rabbit monoclonal, 3174, Cell signalling, 1:200) were diluted in the blocking solution and incubated overnight at 4°C. The next day, coverslips were washed with PBS and incubated with Alexa-conjugated secondary antibodies (1:1000) and DAPI (1:1000) for nuclear staining, in PBS, for 2 hours at room temperature. Following incubation, coverslips were washed again with PBS and mounted using Fluoromount.

Assessing potential for micropeptide production

To evaluate the coding potential of the MYRACL (LINC02488), we first referenced the GENCODE v44 annotation used throughout our snRNA-seq analyses, which classifies this gene as non-coding based on curated experimental and computational evidence. To further validate this, we ran the CPC2 (Coding Potential Calculator 2)[3] on all MYRACL transcripts listed in Ensembl v114. CPC2 evaluates open reading frame structure and composition using four predictive features. The Fickett score measures the positional bias of nucleotides within the RNA sequence, which can indicate whether the sequence is likely to be coding. ORF length is also considered, as protein-coding transcripts typically contain longer and higher-quality open reading frames than non-coding RNAs. ORF integrity assesses whether the identified ORF is structurally valid and complete, further supporting its potential to encode a protein. Lastly, the

isoelectric point (pI) of hypothetical peptides is calculated, based on the premise that peptides derived from non-coding sequences often exhibit different biochemical properties, such as pI, compared to genuine protein-coding sequences. None of the MYRACL transcripts displayed a coding potential score indicative of translation, and no open reading frame greater than 100 amino acids was detected. To explore potential translation of small peptides (sORFs <100 AA), we queried the GWIPS-viz Ribo-seq browser[4], which aggregates ribosome profiling data from 1792 public experiments. No ribosomal coverage indicative of active translation was found at the MYRACL locus. In addition, we manually cross-checked two recently published brain-specific sORF annotations[5,6] and found no annotated translated ORFs overlapping MYRACL. These analyses support the classification of MYRACL as a bona fide lncRNA with no detectable evidence of coding activity across available datasets.

Re-analysis of published single nucleus RNA sequencing datasets

Publicly available single-nucleus RNA sequencing (snRNA-seq) datasets from Jäkel et al.2019[7], the GTEx Consortium[8], Macnair et al.2024[9] were downloaded from the Gene Expression Omnibus (GEO) under accession number GSE118257, the GTex portal, and the European Genome-Phenome Archive (EGA) as dataset EGA: EGAD0000100916 respectively, and the information was correlated with the LncRNA Spatial Atlas of Expression (LncSpA)[10]. Raw FASTQ files were processed using Cell Ranger (v7.2.0) with reference transcriptome incorporating protein-coding and long non-coding RNAs. The reference genome was constructed using cellranger mkref with GENCODE v44, ensuring inclusion of all annotated lncRNA genes. Expression matrices were generated using cellranger count and used as input for downstream analysis. Filtered gene-barcode matrices were imported into Python and processed

using Scanpy (v1.9.6). Cells were filtered based on standard quality control metrics (cells with fewer than 200 detected genes, fewer than 500 total UMI counts, or more than 5% mitochondrial gene expression were excluded from further analysis. Genes detected in fewer than 3 cells were also removed. Gene expression counts were normalized to a total of 10,000 counts per cell. To integrate data across samples and conditions, we employed Seurat v4's reciprocal PCA (RPCA) framework developed by the Satija lab[11]. This method aligns datasets into a shared transcriptional space by identifying anchors across batches while correcting for technical variability. Following integration, we assigned cell type identities based on canonical marker gene expression for oligodendrocyte precursor cells (PDGFRA, CSPG4) and mature oligodendrocytes (MBP, MOG, PLP1). Differential gene expression analysis between annotated OPC and oligodendrocyte clusters was performed using a pseudobulk approach. Specifically, counts from nuclei of the same cell type within each sample were aggregated and used as input for DESeq2. This strategy enables robust detection of biologically meaningful differences while controlling for donor-specific variation. Results were filtered to retain only genes annotated as lncRNAs in GENCODE v44. lncRNAs with log2 fold change ≥ 0.5 and adjusted p-value < 0.05 (Benjamini-Hochberg FDR correction) were considered significantly differentially expressed.

Co-expression analysis

To explore the potential regulatory role of MYRACL in oligodendrocyte lineage development, we performed a targeted co-expression analysis. The expression levels of MYRACL were correlated with all protein-coding genes in mature oligodendrocytes

according to Pearson correlation. Genes with a correlation coefficient $|r| \geq 0.7$ and adjusted $p < 0.05$ were considered significantly co-expressed.

qRT-PCR

RNA was extracted using the RNeasy Mini Kit (Qiagen) following the manufacturer's protocol with minor adjustments. For each sample, 1×10^6 oligodendroglia cells were disrupted in Buffer RLT, mixed with ethanol, and processed through RNeasy spin columns with successive washing steps using Buffers RW1 and RPE. RNA was eluted in RNase-free water and assessed for quality and quantity using a NanoDrop™ Lite spectrophotometer before storage at -80°C. Complementary DNA (cDNA) was synthesized using the iScript™ cDNA synthesis kit (Bio-Rad), with 500 ng RNA reverse-transcribed in a thermocycler under specified conditions indicated by the kit's manufacturer. Quantitative PCR (qPCR) was performed using SsoAdvanced™ Universal SYBR® Green Supermix (Bio-Rad) with UBC as housekeeping gene. Reaction volumes were prepared with primers, cDNA, and nuclease-free water to a total of 10 µl per reaction. Primers were reconstituted into 100 µM stock concentration. The stock was then diluted into 10 µM, and for the qRT-PCR, 1 µL of the 10 µM primer solution was used for a 10 µL reaction volume. Primer efficiencies were determined from standard curves generated by serial cDNA dilutions and calculated using the slope-based equation: $E = 10^{(-1/slope)}$ (**Table S1**). Relative gene expression was analysed using the Pfaffl method, which incorporates individual amplification efficiencies and Ct values to generate accurate expression ratios[12]. The primers used are: UBC: F-TTGAGCCCAGTGACACCATC, R-TTGTAGTCAGACAGGGTGCG, MBP: F-AGCGCACCTGTGATTGATAG, R- AAGACGCGTTTTGGCATCAC, SOX10: F-CCTCACAGATCGCCTACACC, R-CATATAGGAGAAGGCCGAGTAGA, MYRACL:

F-AGTTCAGTTTTATTGGTTGCACGC, R-CTGGTTAGTTTGTAGGGCCTT, OLIG2: F-ATGCACGACCTCAACATCGCCA, R-ACCAGTCGCTTCATCTCCTCCA, PDGFRα: F-GACTTTCGCCAAAGTGGAGGAG, R-AGCCACCGTGAGTTCAGAACGC.

GapmeR mediated knockdown of MYRACL

To perform the transfection and GapmeR knockdown, hESC-derived OPCs are first plated on Day 1 in proliferation medium at a density of 2 × 10^6 cells per well in a 6-well plate or 4 x 10^4 on coverslips. On Day 2, transfection complexes are prepared by diluting 5 μL Lipofectamine-RNAiMAX (ThermoFisher) in Opti-MEM medium (final volume of 500 μL per well for coverslips, or 1 mL per well for 6-well plates) and incubating the mixture at room temperature for 5 minutes. 40 nM of GapmeR was used for the experiments. Equal volumes of the diluted RNAiMAX and GapmeR are then combined (e.g., 500 μL RNAiMAX + 500 μL GapmeR), gently mixed, and incubated at room temperature for 20 minutes to allow transfection complexes to form. During this time, the cells are prepared by removing the growth medium and washing each well with Opti-MEM. Subsequently, the transfection mixture is added to each well, ensuring no pipetting up and down to avoid liposome disruption, and the cells are incubated at 37°C for 6 hours. Following this incubation, one volume of OPC proliferation medium is added to each well, and the cells are returned to 37°C incubation until the next day. On the morning of Day 3, the transfection medium is replaced with one volume of OPC proliferation medium, and the cells are maintained in this condition for 48 hours. On Day 5, the samples are harvested, RNA is extracted, and knockdown efficiency is assessed by qPCR. The following GapmeRs were designed and generated by QIAGEN to target MYRACL (Gap-MY) (Cat. No. LG00790906-GCGTGCAACCAATAAA, and Cat. No. LG00790907-GAAGAATGGCAAACGC).

Lentiviral overexpression of MYRACL transcripts

In order to overexpress MYRACL within the hESC-derived oligodendroglia, 3rd generation lentiviruses including MYRACL exonic sequence, (Ensembl annotation GRCh38.p13) was generated by the Biomolecular Core Facility of the University of Edinburgh, UK. To perform the transduction, hESC-derived OPCs are first plated on Day 1 in proliferation medium at a density of 2×10^6 cells per well in a 6-well plate or 4×10^4 on coverslips. On Day 2, infection complexes are prepared by diluting 0.5 µL Polybrene (ThermoFisher) in Opti-MEM medium (final volume of 500 µL per well for coverslips, or 1 mL per well for 6-well plates). The generated lentiviruses were used at 40 MOI and were diluted into the polybrene mix and then were added to the cells. The cells are incubated at 37°C overnight. Following this incubation, one volume of OPC proliferation medium is added to each well, and the cells are returned to 37°C incubation until the next day. On the morning of Day 3, the transfection medium is replaced with one volume of OPC proliferation medium, and the cells are maintained in this condition for 48 hours. On Day 5, the samples are harvested, RNA is extracted, and overexpression efficiency is assessed by qPCR.

Myelination assay on organotypic Shiverer brain sections ex vivo

The generation of organotypic Shiverer brain sections and the myelination assay was performed as previously described[13]. Briefly, P0–P2 Shiverer pups were euthanized following the standard UK Home Office regulations under project licences PADF15B79 and PP1335335, and their brains were extracted and placed in cold Hibernate™-A medium (Thermo Scientific) on ice. The brains were mounted on a vibratome (LEICA) and coronal cortical slices (250–300 µm) were prepared in cold Hibernate™-A medium and transferred to Millicell cell culture inserts (Merck-Millipore). The slices were

cultured in a warm medium containing 50% MEM (Life Technologies), 25% Earle's Balanced Salt Solution (Life Technologies), 25% heat-inactivated horse serum (Thermo Scientific), 1% Glutamax™ supplement (Thermo Scientific), 1% penicillin–streptomycin, 0.5% Amphotericin B (Thermo Scientific), and 6.5 mg/ml glucose (Sigma-Aldrich).

Slices were maintained in serum-containing medium and then transitioned to serum-free medium containing DMEM/F12 (Thermo Scientific), 1% B-27™ supplement, 0.5% N2 supplement, 1% Glutamax™ supplement, 1% penicillin–streptomycin, and 0.5% Amphotericin B. All cultures were incubated at 37°C and 5% CO2, with medium changes every two days. After 7 days in culture, 100,000 hESC-derived OPCs cells were seeded onto each cortical slice and co-cultured for an additional four weeks. Then, slices were washed once with 1x PBS before being fixed in 4% paraformaldehyde (PFA) for 1 hour at room temperature. After fixation, the slices were rinsed in 1x PBS and blocked for 2 hours at room temperature in a solution containing 3% heat-inactivated horse serum, 2% BSA (Sigma-Aldrich, A7906), and 0.5% Triton X-100 in 1x PBS. Following the blocking step, the slices were incubated at 4°C for 48 hours with primary antibodies (anti-MBP, rat monoclonal, MCA409S, BioRad, 1:250), anti-Neurofilament-H (chicken polyclonal, 822601, BioLegend, 1:100), anti-Caspr (rabbit polyclonal, ab34151, Abcam, 1:1000) diluted in the blocking solution. After primary antibody incubation, slices were washed three times with blocking solution and then incubated overnight at 4°C with the appropriate secondary antibodies. Finally, the slices were washed in 1x PBS, counterstained with Hoechst 33342 solution, and mounted onto glass microscope slides using Fluoromount.

Subcellular cellular fractionation

Isolation of the nuclear and cytoplasmic fragments of hESC-derived OPCs and oligodendrocytes was performed using the PARIS™ Kit (Invitrogen, AM1921) according to manufacturer's instructions. For the cellular fractionation experiments, triplicates of $5*10^5$ hESCs, $6*10^6$ OPCs and $6*10^6$ Oligodendrocytes were used.

RNAscope

To localize expression of MYRACL within the human oligodendroglia, RNAscope was performed in human ESC-derived oligodendroglia cultures using the RNAscope™ Multiplex Fluorescent Reagent Kit v2 (ACD), according to manufacturer's instructions using a customized probes specifically binding to MYRACL and OPALIN transcripts (RNAscope™ Probe- Hs-OPALIN, Cat No. 579861, RNAscope™ Probe- Hs-MYRACL-C2, Cat No. 1585881-C2, RNAscope™ Probe- Hs-MYRACL-C1, Cat No. 1585871-C1).

Statistical analysis

The experimenter was blinded to the experimental conditions when performing imaging and analysis of data. Data were checked for normality and parametric tests were used for statistical analysis. One-way ANOVA followed by Bonferroni multiple comparisons correction or Student's t-Test were performed using GraphPad Prism 10. Data presented as means +/- SEM with data points of individual biological replicates. Illustrations created with BioRender.com.

Supplemental references

1. Wagstaff, L.J., Bestard-Cuche, N., Kaczmarek, M., Fidanza, A., McNeil, L., Franklin, R.J.M., and Williams, A.C. (2024). CRISPR-edited human ES-derived oligodendrocyte progenitor cells improve remyelination in rodents. Nature Communications *15*, 8570. 10.1038/s41467-024-52444-w.
2. Livesey, M.R., Magnani, D., Cleary, E.M., Vasistha, N.A., James, O.T., Selvaraj, B.T., Burr, K., Story, D., Shaw, C.E., Kind, P.C., et al. (2016). Maturation and electrophysiological properties of human pluripotent stem cell-derived oligodendrocytes. Stem Cells *34*, 1040-1053. 10.1002/stem.2273.
3. Kang, Y.J., Yang, D.C., Kong, L., Hou, M., Meng, Y.Q., Wei, L., and Gao, G. (2017). CPC2: a fast and accurate coding potential calculator based on sequence intrinsic features. Nucleic Acids Res *45*, W12-w16. 10.1093/nar/gkx428.
4. Michel, A.M., Fox, G., M. Kiran, A., De Bo, C., O'Connor, P.B.F., Heaphy, S.M., Mullan, J.P.A., Donohue, C.A., Higgins, D.G., and Baranov, P.V. (2013). GWIPS-viz: development of a ribo-seq genome browser. Nucleic Acids Research *42*, D859-D864. 10.1093/nar/gkt1035.
5. Duffy, E.E., Finander, B., Choi, G., Carter, A.C., Pritisanac, I., Alam, A., Luria, V., Karger, A., Phu, W., Sherman, M.A., et al. (2022). Developmental dynamics of RNA translation in the human brain. Nat Neurosci *25*, 1353-1365. 10.1038/s41593-022-01164-9.
6. Chothani, S.P., Adami, E., Widjaja, A.A., Langley, S.R., Viswanathan, S., Pua, C.J., Zhihao, N.T., Harmston, N., D'Agostino, G., Whiffin, N., et al. (2022). A high-resolution map of human RNA translation. Molecular Cell *82*, 2885-2899.e2888. https://doi.org/10.1016/j.molcel.2022.06.023.
7. Jäkel, S., Agirre, E., Mendanha Falcão, A., van Bruggen, D., Lee, K.W., Knuesel, I., Malhotra, D., ffrench-Constant, C., Williams, A., and Castelo-Branco, G. (2019). Altered human oligodendrocyte heterogeneity in multiple sclerosis. Nature *566*, 543-547. 10.1038/s41586-019-0903-2.
8. Consortium, T.G., Aguet, F., Anand, S., Ardlie, K.G., Gabriel, S., Getz, G.A., Graubert, A., Hadley, K., Handsaker, R.E., Huang, K.H., et al. (2020). The GTEx Consortium atlas of genetic regulatory effects across human tissues. Science *369*, 1318-1330. doi:10.1126/science.aaz1776.
9. Macnair, W., Calini, D., Agirre, E., Bryois, J., Jäkel, S., Smith, R.S., Kukanja, P., Stokar-Regenscheit, N., Ott, V., Foo, L.C., et al. (2025). snRNA-seq stratifies multiple sclerosis patients into distinct white matter glial responses. Neuron *113*, 396-410.e399. 10.1016/j.neuron.2024.11.016.
10. Lv, D., Xu, K., Jin, X., Li, J., Shi, Y., Zhang, M., Jin, X., Li, Y., Xu, J., and Li, X. (2020). LncSpA: LncRNA Spatial Atlas of Expression across Normal and Cancer Tissues. Cancer Res *80*, 2067-2071. 10.1158/0008-5472.Can-19-2687.
11. Hao, Y., Stuart, T., Kowalski, M.H., Choudhary, S., Hoffman, P., Hartman, A., Srivastava, A., Molla, G., Madad, S., Fernandez-Granda, C., and Satija, R. (2024). Dictionary learning for integrative, multimodal and scalable single-cell analysis. Nature Biotechnology *42*, 293-304. 10.1038/s41587-023-01767-y.
12. Pfaffl, M.W. (2001). A new mathematical model for relative quantification in real-time RT-PCR. Nucleic Acids Res *29*, e45. 10.1093/nar/29.9.e45.

13. Tsarouchas, T.M., Zoupi, L., Williams, A., and Gibson, E.M. (2025). Protocol for assessing myelination by human iPSC-derived oligodendrocytes in Shiverer mouse ex vivo brain slice cultures. STAR Protoc *6*, 103609. 10.1016/j.xpro.2025.103609.
