# Supplementary material for: The lncRNA MYRACL regulates human oligodendrocyte maturation and myelination
Source: Mol Ther. 2025 Aug 8;33(12):6025–32. doi: 10.1016/j.ymthe.2025.08.011 (PMC12703153; doi:10.1016/j.ymthe.2025.08.011)
Supplement: Document S2. Article plus supplemental information [file mmc3.pdf]

Molecular Therapy
Brief report

The lncRNA *MYRACL* regulates human oligodendrocyte maturation and myelination

Themistoklis M. Tsarouchas,[1,2,9] Francesca Vacante,[3,8,9] Nina-Lydia Kazakou,[1,4] Laura Wagstaff,[1] Matthew Bennett,[3] Lida Zoupi,[5,6] Erin M. Gibson,[2] Andrew H. Baker,[3,7] and Anna Williams[1]

[1]Centre for Regenerative Medicine, Institute for Regeneration and Repair, University of Edinburgh, Edinburgh BioQuarter, EH16 4UU Edinburgh, UK; [2]Department of Psychiatry and Behavioral Sciences, Stanford University School of Medicine, Palo Alto, CA 94305, USA; [3]Queens Medical Research Institute, British Heart Foundation Centre for Research Excellence, Centre for Cardiovascular Sciences, University of Edinburgh, EH16 4TJ Edinburgh, UK; [4]Novo Nordisk Foundation for Stem Cell Medicine, reNEW, University of Copenhagen, Copenhagen 2200, Denmark; [5]Centre for Discovery Brain Sciences, The University of Edinburgh, EH8 9XD Edinburgh, UK; [6]Simons Initiative for the Developing Brain, University of Edinburgh, EH8 9XD Edinburgh, UK; [7]Department of Pathology, Cardiovascular Research Institute Maastricht School for Cardiovascular Diseases, Maastricht University, 6229 ER Maastricht, the Netherlands

Recent studies have described disease-associated expression patterns of long non-coding RNAs (lncRNAs) associated with neurodevelopment and neurodegeneration, highlighting their potential as regulators of function and therefore potential therapeutic targets. Oligodendrocyte (OL) dysfunction drives central nervous system myelin disruption in neurological disorders, but the mechanisms underlying impaired myelin patterns are still poorly understood. In this study, we uncover a role for the lncRNA MYRACL (myelination regulating oligodendrocyte-associated lncRNA) as a regulator of functional maturation and OL myelination. Analysis of RNA-sequencing data performed in human postmortem brain tissue revealed MYRACL to be among the top enriched genes expressed in the OL population compared to the OL precursor cell cluster. We validated this finding in an embryonic stem cell-derived oligodendroglia cell culture model. Analysis of evolutionary conservation and protein coding potential showed that MYRACL is non-coding and may exhibit conserved regions across mammalian species. Further co-expression analysis of lncRNAs-mRNAs suggested that expression of MYRACL positively correlates with genes known to be involved in driving oligodendroglia differentiation. GapmeR-mediated knockdown of nuclear MYRACL disrupted OL maturation *in vitro*, while lentivirus-mediated overexpression promoted OL differentiation with enhancement of myelin formation *in vitro*. Our findings highlight MYRACL as a novel regulatory mechanism in human OL maturation and myelination. By providing a human, translationally relevant platform, this work advances our ability to model human myelination *in vitro* and paves the way for precision medicine approaches targeting lncRNA-mediated dysregulation in neurodevelopmental and neurodegenerative diseases.

INTRODUCTION

Oligodendrocyte (OL) differentiation is crucial for central nervous system (CNS) function, particularly in the formation of myelin, which enables rapid and efficient electrical signal conduction along axons.[1] This complex process involves a tightly regulated interplay of signaling pathways, growth factors, and transcription factors that collectively guide the maturation of OL precursor cells (OPCs) into myelinating OLs.[2] A two-tier transcriptional control mechanism has been described, wherein epigenetic repression of inhibitory genes coincides with activation of myelin-related gene expression to drive differentiation.[3] Given that OPCs persist into adulthood and can respond to injury or disease, disruptions in their differentiation are increasingly being recognized as contributing factors to neurodegenerative and psychiatric disorders.[1] Amid growing interest in the regulatory complexity of CNS development, long non-coding RNAs (lncRNAs) have emerged as important modulators of gene expression.[4] Approximately 40% of all lncRNAs are enriched in the brain.[5,6] Examples include *OLMALINC* and lnc-*PINT*, two conserved nuclear lncRNAs involved in OL maturation and the regulation of cell proliferation, oxidative stress, and apoptosis, respectively.[7,8] Additionally, the lncRNAs *Gomafu* and *Neat1* have been shown to be expressed throughout the oligodendroglia lineage progression and appear to be upregulated during OL lineage specification and maturation.[9] Similarly, lncRNAs *PNKY* and *PAUPAR* play critical roles in neurogenesis, shaping transcriptional networks and chromatin dynamics to guide neural stem cell differentiation and cortical development.[5,10] lncRNAs have also been implicated in neurological disorders characterized by aberrant myelination. For example, *BACE1-AS*, a circulating biomarker in Alzheimer

Received 21 April 2025; accepted 5 August 2025;
https://doi.org/10.1016/j.ymthe.2025.08.011.

[8]Present address: Stanford Cardiovascular Institute, School of Medicine, Stanford, CA 94305, USA

[9]These authors contributed equally

Correspondence: Andrew H. Baker, Queens Medical Research Institute, British Heart Foundation Centre for Research Excellence, Centre for Cardiovascular Sciences, University of Edinburgh, EH16 4TJ Edinburgh, UK.
E-mail: andy.baker@ed.ac.uk
Correspondence: Anna Williams, Centre for Regenerative Medicine, Institute for Regeneration and Repair, University of Edinburgh, Edinburgh BioQuarter, EH16 4UU Edinburgh, UK.
E-mail: anna.williams@ed.ac.uk

Published by Elsevier Inc. on behalf of The American Society of Gene and Cell Therapy.
This is an open access article under the CC BY license (http://creativecommons.org/licenses/by/4.0/).

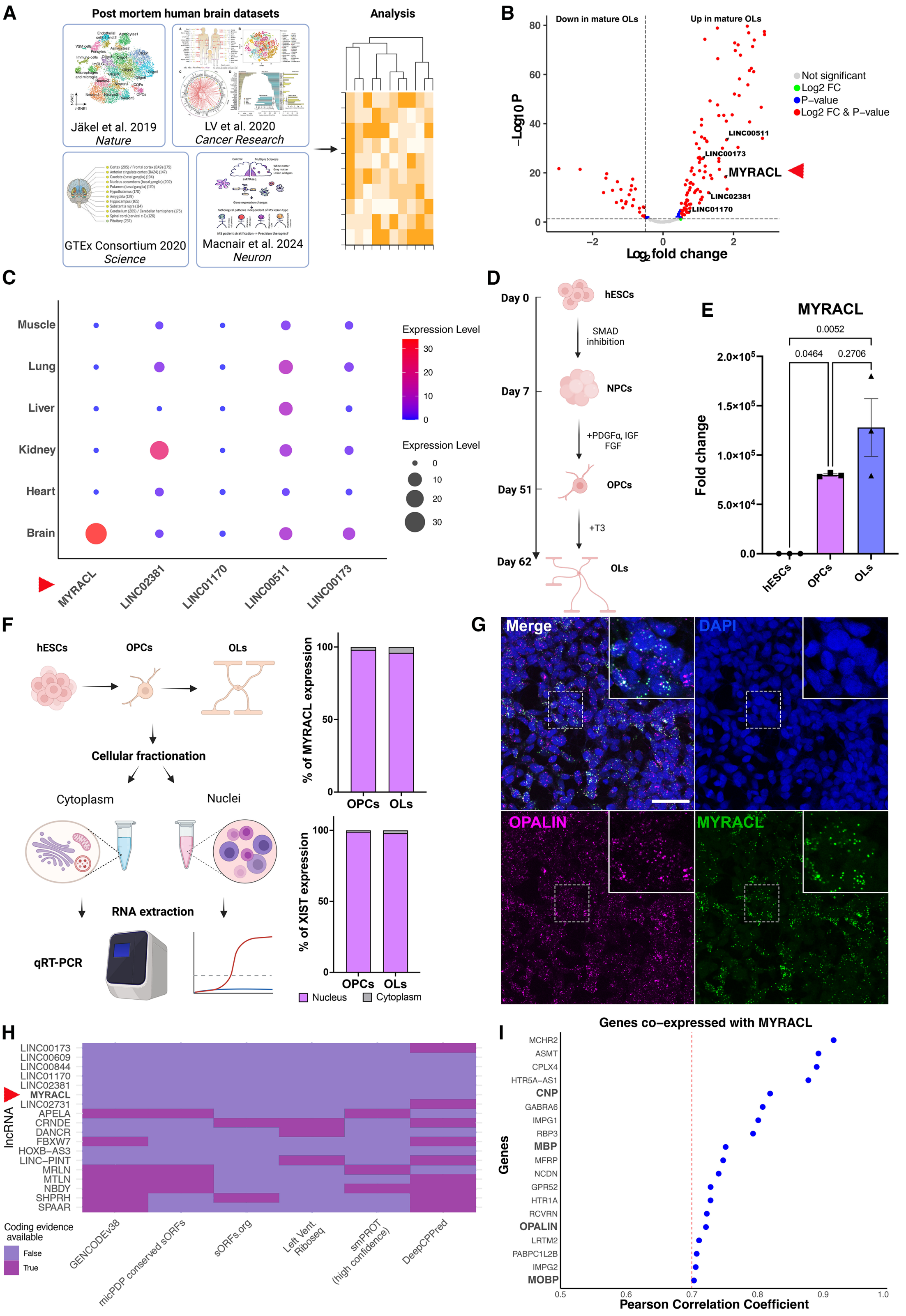

(legend on next page)

disease (AD), stabilizes *BACE1* mRNA leading to enhanced Aβ amyloid deposition in AD.[11] Additionally, lncRNAs are known for regulating microRNAs in health and disease.[12] Examples include *KCNQ1OT1* and *SNHG1*, which control NLRP3 inflammasomes via the regulation of miR-30e-3p and miR-7, respectively, in multiple sclerosis (MS)[13] and Parkinson disease (PD).[14] In murine OLs, lnc*OL1* interacts with SUZ12 and regulates myelination in the developing brain.[15] Additionally, lnc-*OPC* has been found to be regulated by OLIG2[16] in the mouse brain, and this interaction is essential for OPC generation, whereas the lncRNA *TubAR* has been shown to interact with TUBB4A and TUBA1A to facilitate microtubule assembly and support of the maintenance of myelination.[17] This evidence highlights the value of lncRNAs as potential therapeutic targets for neurological disorders.

Despite these insights, the role of lncRNAs in human oligodendroglia development, differentiation, and myelination remains largely unknown, primarily due to the decreased conservation between mouse and human lncRNAs[18] and the limited functional human data and reliable human models that can accurately recapitulate human oligodendroglial biology. Human embryonic stem cells (hESCs) are a powerful tool for the study of development and maturation processes as well as pro-pathological phenotypes, providing a valuable platform to effectively assess mechanisms controlling cell physiology and disease.[19] In this study, using *in vitro* cultures of ESC-derived OPCs and OLs combined with *ex vivo* mouse cultures, we identified *MYRACL* (myelination regulating oligodendrocyte-associated lncRNA), a nuclear brain-specific long intergenic RNA (lincRNA), as a regulator of human OL maturation and myelination.

RESULTS

MYRACL is a CNS-specific lncRNA enriched during the differentiation of human OLs

To identify lncRNAs whose expression pattern is altered during oligodendroglia differentiation, we re-analyzed publicly available relevant single-nucleus RNA sequencing (snRNA-seq) and spatial transcriptomics datasets from human postmortem brain tissues, encompassing transitions from OPCs to mature OLs (Figure 1A). Using non-coding annotations present in the reference transcriptome, this analysis identified *MYRACL, LINC00173, LINC02381, LINC00511*, and *LINC01170* among the top enriched lncRNAs during this transition (Figure 1B; Table S2). As lncRNAs frequently exhibit tissue-specific expression patterns, we leveraged LncSpA, a tissue-specific atlas of human lncRNAs.[20] This pinpointed *MYRACL* as a highly expressed lncRNA in brain tissue compared to other identified lncRNAs (Figure 1C). *MYRACL* is a lincRNA, currently annotated as *LINC02488* (ENSEMBL: ENSG00000 249362), located in position chr5:87,607,623–87,933,811 of the human genome (GENCODE version 47). By looking at the syntenic region of the mouse genome, we have also identified two regions proximal to the transcriptional start site with elevated phyloP scoring (mean of 1.36 for mm10:chr13:84,931,280–84,931,648 and mean of 1.85 for mm10:chr13:84,933,017–84,933,225), suggesting potential conservation across placental mammals. To further validate the *MYRACL* expression profile, we used an established *in vitro* differentiation platform in which hESCs are directed to differentiate into OPCs and subsequently into OLs within a period of 62 days (Figure 1D). Quantitative reverse transcription PCR (RT-qPCR) analysis showed a progressive increase in *MYRACL* expression during the differentiation process (Figure 1E). We validated the expression patterns of all identified lncRNAs during *in vitro* differentiation and observed that only *MYRACL* exhibited consistent upregulation during the transition from OPCs to OLs (data not shown). These results directed our focus on the study of *MYRACL* as a brain-specific lncRNA. To gain insights into the cellular localization of *MYRACL*, which can provide information on its potential cellular function, we performed fractionation of human oligodendroglia and found that *MYRACL* predominantly localized in the nucleus of both hESC-derived OPCs and OLs (Figure 1F). We used *XIST*, a well-characterized nuclear lncRNA, as a positive control to confirm accuracy of fractionation (Figure 1F). To gain information on the spatial localization of *MYRACL*, we performed RNA-Scope *in situ* hybridization on hESC-derived oligodendroglia. *MYRACL* showed robust expression in mature OLs, as evidenced by its co-localization with the OL marker *OPALIN*, with cytoplasmic localization (Figure 1G). As lncRNAs are often re-annotated as coding genes after uncovering evidence of micropeptide production,[21] we checked this possibility for *MYRACL* using available resources that assess this possibility.[22,23] *MYRACL* and other OL-enriched lncRNAs still lacked any evidence of coding potential compared to genes with strong evidence of micropeptide production, such as *SPAAR*,[24] *DANCR*,[25] and *APELA*[26] (Figures 1H; Table S4). Correlation analysis of *MYRACL* expression with other known regulators of oligodendroglia dynamics enriched in OPC to OL differentiation, combined with the information

Figure 1. *MYRACL* is a brain-specific nuclear lncRNA upregulated in mature oligodendrocytes

(A) Schematic representation of the pipeline used to re-analyze three published snRNA-seq datasets from postmortem brain tissue and correlated with the LncSpA atlas. (B) Volcano plot displaying the top differentially expressed lncRNAs in mature oligodendrocytes (OLs) versus OPC stage. (C) Bubble plot showing tissue-specific expression profiles for the top enriched lncRNAs identified. (D) Schematic of hESC-derived OPC and OL differentiation protocol (62 days). (E) RT-qPCR analysis showing increased *MYRACL* expression moving from hESCs to OPCs to OLs ($n = 3$), one-way ANOVA ($F(2,6) = 8.946$) followed by Bonferroni multiple comparisons. (F) Schematic and RT-qPCR data of *MYRACL* expression showing more nuclear location compared to cytoplasmic regions following cellular fractionation of hESC-derived OPCs and OLs. *XIST*, a well-characterized nuclear lncRNA, served as a positive control for nuclear enrichment ($n = 1$). (G) RNA-Scope *in situ* hybridization on hESC-derived oligodendroglia. *MYRACL* is expressed by *OPALIN*$^+$ OLs. Dashed rectangles indicate a representative area within the culture that is visualized in higher magnification on the top right of the panel. (H and I) Scale bar, 50 μm. (H) Evidence of the non-coding status of *MYRACL* and other OL-enriched lncRNAs in selected databases relevant for coding potential and/or micropeptide production alongside positive controls of previously annotated lncRNAs with strong evidence of micropeptide production. (I) Co-expression analysis of *MYRACL* showing positive correlation with OL markers *MBP, MOBP, OPALIN,* and *CNP* enriched in OPC to OL differentiation (Pearson correlation >0.7).

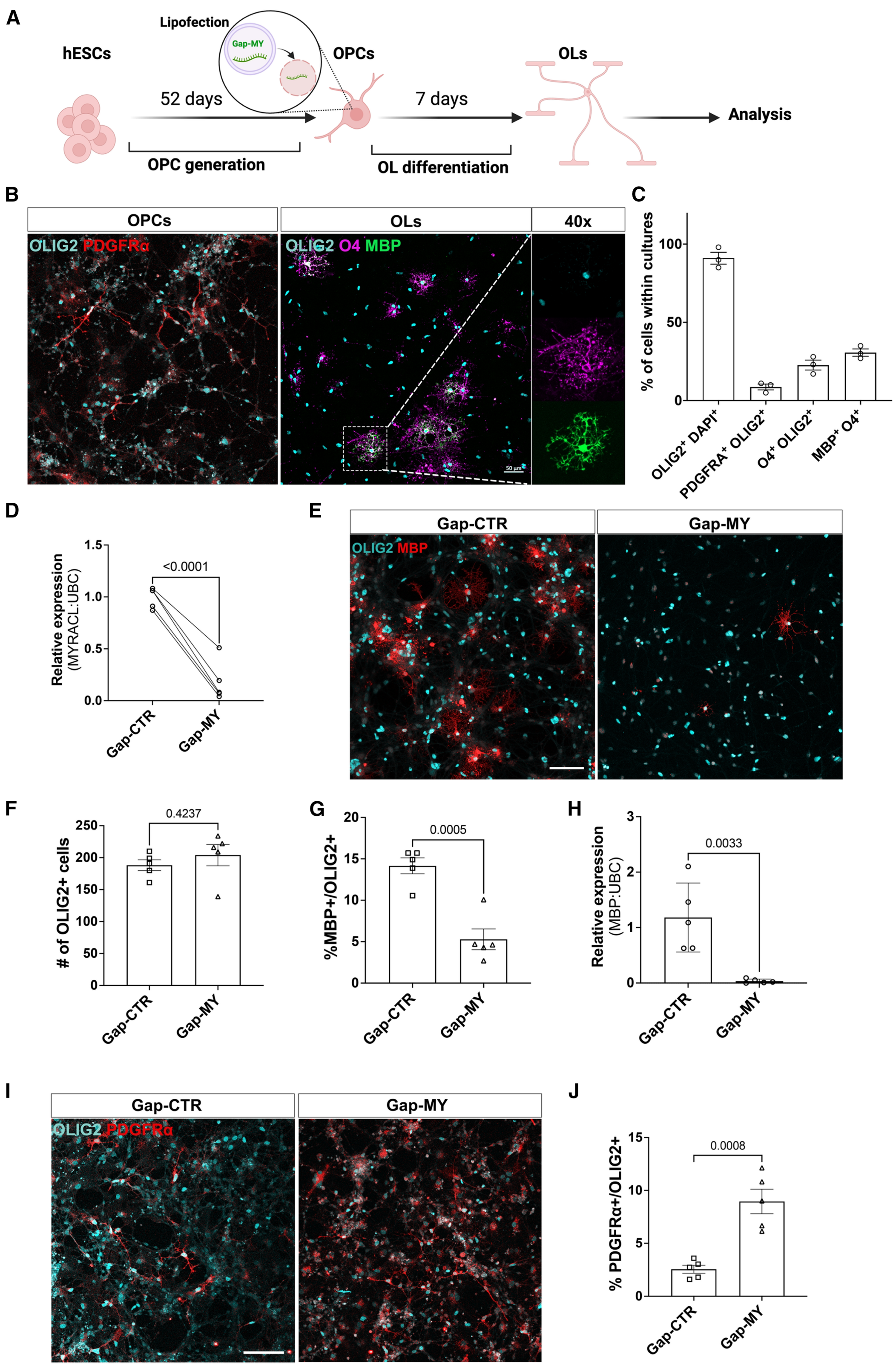

(legend on next page)

from the LncSPA atlas, showed a positive correlation between *MYRACL* and OL markers, within the three datasets, including *MBP, OPALIN, MOBP*, and *CNP* (Figures 1I; Table S3).

Depletion of *MYRACL* impairs human OPC to OL differentiation *in vitro*

To assess the role of *MYRACL* in human oligodendroglia differentiation and myelination, we performed depletion of its expression using GapmeR technology in OPC to OL differentiation (Figures 2A–2C). We first evaluated the GapmeR-mediated knockdown efficiency and confirmed significant downregulation of *MYRACL* following transfection with *MYRACL* GapmeR (Gap-MY) compared to control (Gap-CTR) (Figure 2D). We then assessed the functional consequence of *MYRACL* depletion by assessing oligodendroglia generation and OL differentiation through immunofluorescence (IF) staining and RT-qPCR for OLIG2 and myelin basic protein (*MBP*), respectively. While the number of OLIG2$^+$ cells was not affected following treatment with Gap-MY, indicating no effects on the generation of oligodendroglial lineage cells, the depletion of *MYRACL* led to decreased levels of *MBP* at the transcriptional and MBP$^+$ cell density levels (Figures 2E–2H). Additionally, *MYRACL*-depleted cells exhibited a significant increase in the proportion of platelet-derived growth factor receptor α$^+$ (PDGFRα$^+$)/OLIG2$^+$ cells (Figures 2I and 2J). As PDGFRα is a marker of the OPC stage, these results suggest a decrease in the maturation progression toward mature OLs.

Overexpression of *MYRACL* regulates human OL differentiation and myelination *in vitro* and *ex vivo*

Given that depletion of *MYRACL* decreases the differentiation of human OLs, we next sought to understand the functional consequences of *MYRACL* overexpression. To efficiently enhance its expression, we generated a lentiviral vector (LV) carrying the lncRNA transcript (*GRCh38.p13*) named LV-MY. hESC-derived OPCs were transduced with either LV-MY or an empty control lentivirus (LV-CTR) for 48 h (Figure 3A). Lentiviral transduction efficiency was then assessed by RT-qPCR analysis, confirming overexpression of the lncRNA compared to the control lentivirus (Figure 3B). To determine the functional consequence of *MYRACL* overexpression on OL maturation, we examined the expression of key OL lineage markers, including *SOX10*, *OLIG2*, *PDGFRα*, and *MBP*, by RT-qPCR analysis. Lentiviral-mediated overexpression resulted in a significant upregulation of *SOX10* and *OLIG2*, two critical transcription factors required for OL differentiation, along with an increase in *MBP* expression, indicating enhanced progression to the myelinating stage. In contrast, LV-02488 did not alter the expression of the OPC marker PDGFRα compared to control (Figures 3C–3F). To assess the effects of *MYRACL* overexpression on myelination, transduced OPCs were transplanted onto organotypic brain slice cultures derived from *Shiverer (MBP$^{shi/shi}$)* mice, a model that lacks functional MBP and thus lacks compact myelin due to a mutation in the *MBP* gene (Figure 3A). We leveraged the *Shiverer* brain slices *ex vivo* culture model[27] because MBP$^+$ myelin formed in these cultures is by definition derived from hESC-derived OPCs and because the three-dimensional structure is a more physiologically relevant environment. At 4 weeks post-transplantation, overexpression of *MYRACL* enhanced MBP protein expression and myelin sheath formation, as shown by the quantification of MBP coverage normalized to the axonal area. Colocalization of MBP and contactin-associated protein (CASPR)[28] at the myelin segments indicates the generation of myelin sheaths after the overexpression of *MYRACL* (Figures 3G–3J). However, the total number of MBP$^+$ cells was unaffected, indicating that the overexpression of *MYRACL* altered the amount of myelin sheaths rather than OL number (Figure 3K). This was not dependent on axon availability, which remained unchanged across conditions (Figure 3L).

Therefore, the above data from gain- and loss-of-function experiments in human oligodendroglia indicate that *MYRACL* is important for differentiation and myelin sheath formation.

DISCUSSION

Given their broad regulatory roles in cellular processes, lncRNAs are increasingly recognized as key modulators of oligodendroglial dynamics and differentiation within the CNS.[15–17,29] In this study, we identified *MYRACL* as a novel candicate lncRNA that plays an important role in the maturation of human OLs and the process of myelination *in vitro*. In addition to OLs, *MYRACL* shows detectable expression in inhibitory neurons, while its expression is absent in excitatory neurons, astrocytes, and microglia. Our data provide insights into *MYRACL* expression profiles throughout human oligodendroglia differentiation, tissue-specific spatial expression in human biospecimens, coding potential, and subcellular localization. We show that *MYRACL* expression increases during the differentiation of human OLs, suggesting its involvement in the maturation process. We were able to effectively modulate the expression of *MYRACL* in gain- and loss-of-function studies, showing that *MYRACL* overexpression promoted OL differentiation and

Figure 2. GapmeR-mediated *MYRACL* knockdown decreases MBP expression while increasing PDGFRα-expressing cells

(A) Schematic of *in vitro* depletion of *MYRACL* during the hESC-OPC to OL differentiation process. (B) Example micrographs of the generated OPCs and OLs generated *in vitro*. The dashed rectangle indicates a selected OL that expresses OLIG2, O4, and MBP and is visualized in higher magnification on the right side of the panel. (C) Quantification of *in vitro* differentiation efficiency ($n = 3$). (D) RT-qPCR of *MYRACL* following Gap-MY treatment of OPC versus Gap-CTR following 7 days of treatment ($n = 5$), unpaired two-tailed t test ($t = 8.285$, $df = 8$). (E) Representative photomicrographs of OLIG2$^+$/MBP$^+$ oligodendroglial cells after Gap-MY treatment or control (scale bar, 20 μm) ($n = 5$). (F and G) Quantification of IF staining of OLIG2$^+$ cells, unpaired two-tailed t test ($t = 0.8431$, $df = 8$), mature OLs (OLIG2$^+$/MBP$^+$ cells) ($n = 5$), and unpaired two-tailed t test ($t = 5.624$, $df = 8$). (H) RT-qPCR of *MBP* following treatment with Gap-MY or control ($n = 5$), unpaired two-tailed t test ($t = 3.574$, $df = 8$). (I and J) Micrographs of OLIG2$^+$/PDGFRα$^+$ in *MYRACL*-depleted OLs or control and quantification of cell densities ($n = 5$), unpaired two-tailed t test ($t = 5.261$, $df = 8$). All Scale bars, 50 μm. Bars show mean with SEM for all and p values given on graphs.

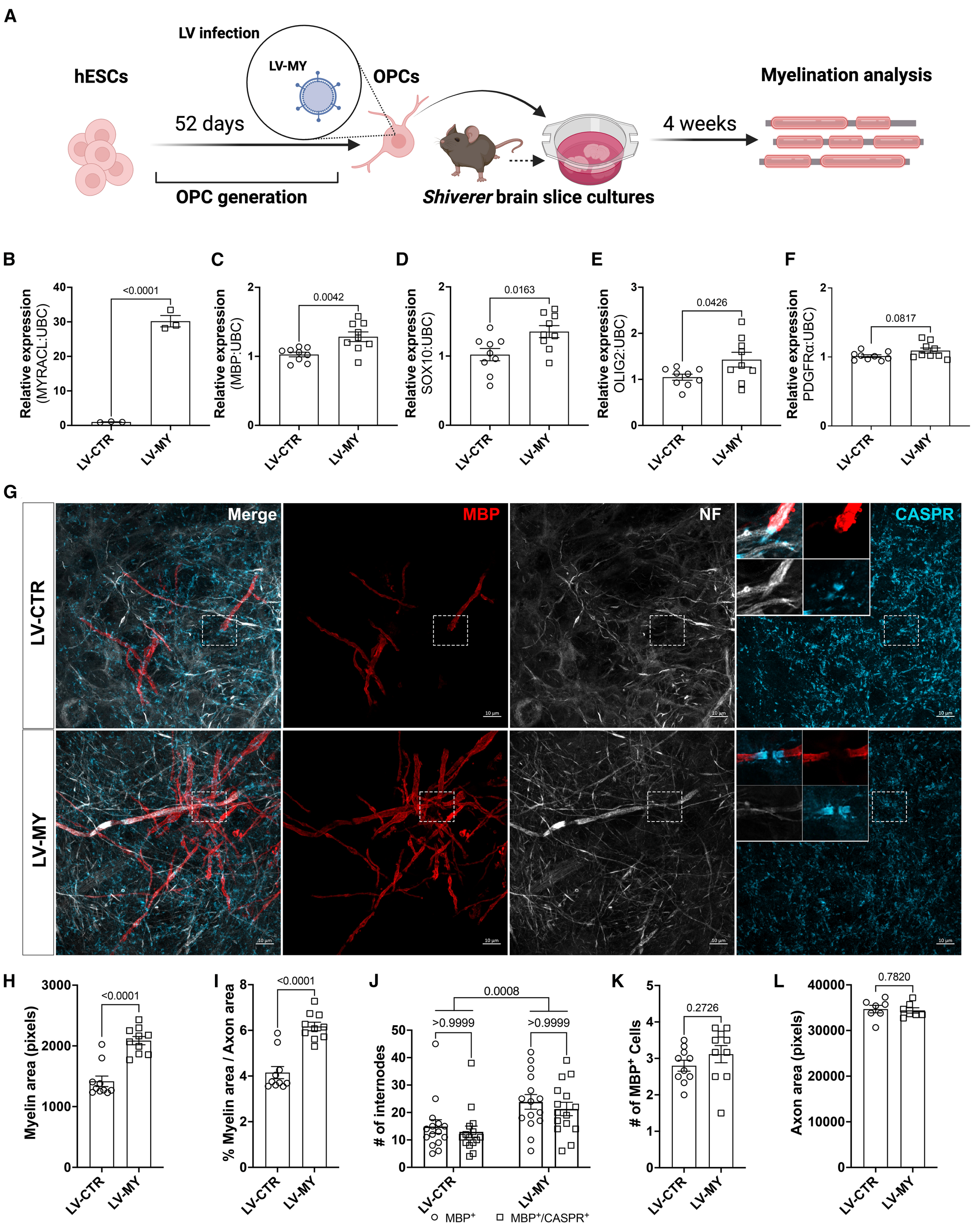

(legend on next page)

enhanced myelin formation *in vitro*, whereas depletion impaired OL differentiation.

The nuclear localization of *MYRACL* suggests possible roles associated with epigenetic or post-transcriptional regulation of gene expression.[4–6] Given the established role of lncRNAs in tuning gene expression during neural development,[5] we conducted a co-expression analysis to explore the potential pathways in which *MYRACL* may be functionally involved. The results showed a positive correlation between *MYRACL* and the canonical markers of mature OLs *MBP, MOBP, CNP,* and *OPALIN*. While this might suggest potential involvement in similar pathways, further validation studies are needed to confirm the functional relevance of these associations. Additionally, we found that overexpressing *MYRACL* led to increased expression of MBP, SOX10, and OLIG2 while PDGFRα levels remained unchanged. *MBP* expression was also positively correlated with *MYRACL* levels (Figure 1I), but no such correlation was seen for *SOX10, OLIG2*, or *PDGFRA*. These results suggest that the lncRNA may support the transition toward a more mature OL state, rather than affecting early lineage specification. It is not yet known whether a murine *MYRACL* homolog exists with a similar function or whether this is a species-specific regulatory pathway.

Understanding the mechanisms by which *MYRACL* controls OL maturation could provide new insights into therapeutic strategies for neurological disorders characterized by aberrant myelination in development such as in autism and by demyelination (needing remyelination) such as in MS and AD. We identified *MYRACL* from RNA-seq datasets of control and MS donors, but whether *MYRACL* expression is altered in other diseases with myelin deficit is unknown and could provide additional insights into to whether lncRNAs could act as a general target for promoting (re)myelination and restoring neural function in disease.[4,7,13,15,17] Additionally, overexpression of *MYRACL* to improve the efficiency of human *in vitro* differentiation methods may be useful in assays for regenerative medicine research.

Although lentiviral overexpression of genes is efficient for functional research, its genomic integration presents challenges for therapeutic applications in humans. Alternate approaches, including non-integrating systems such as adeno-associated virus or lipid nanoparticles, or CRISPR-based endogenous genomic regulatory element activation, could provide more clinically relevant means of altering *MYRACL* expression. Mechanistic studies are required to dissect the specific role of *MYRACL* within the oligodendroglial transcriptional network, particularly in relation to key myelin-associated genes such as *MBP, MOBP, OPALIN*, and *CNP*, which may act either as targets, co-regulated partners, or downstream effectors. Understanding how *MYRACL* interfaces with these canonical markers will be essential for delineating its contribution to the molecular architecture of myelination.

In conclusion, we identified *MYRACL* as a novel regulator of human OL maturation and myelination, which may enhance our understanding of human myelin disorders and pave the way for targeted therapeutic strategies.

MATERIALS AND METHODS

This information can be found in the supplemental information.

DATA AVAILABILITY

Data are included within the paper. Raw data collected are available from the corresponding authors upon request.

ACKNOWLEDGMENTS

The authors wish to thank Dr. Pamela Brown (IRR Biomolecular Core, University of Edinburgh) for her technical assistance generating the viral vectors used in this study. This study was supported by the Multiple Sclerosis (MS) Society UK (A.W.), the Medical Research Council (MRC) (MR/P016022/1) (A.W.), the MRC-MS Society UK (MR/T015594/1) (A.W.), the British Heart Foundation (BHF) (ReGenLnc) (A.H.B.), British Heart Foundation Programme grant no. RG/20/5/34796 (A.H.B.), and the BHF Chair of Translational Cardiovascular Sciences, Wellcome Trust iTPA PIII-020SF (T.M.T. and F.V.). For the purpose of open access, the author has applied a Creative Commons Attribution (CC BY) license to this manuscript.

AUTHOR CONTRIBUTIONS

Conceptualization and methodology, T.M.T., F.V., A.H.B., and A.W. Investigation and analysis, T.M.T., F.V., N.-L.K., L.W., M.B., and L.Z. Writing – original draft, T.M.T. F.V., A.H.B., and A.W. Writing – review & editing, T.M.T., F.V., N.-L.K., L.W., M.B., L.Z., E. M.G., A.H.B., and A.W. Visualization, T.M.T. and F.V. Funding acquisition, T.M.T., F. V., A.H.B., and A.W. Supervision, A.H.B. and A.W.

DECLARATION OF INTERESTS

The authors declare no competing interests.

Figure 3. Lentiviral-mediated overexpression of *MYRACL* enhances human OL differentiation and myelination

(A) The experimental setup of lentiviral transduction of OPC cultures and subsequent transplantation onto *Shiverer* brain slice cultures for myelination assessment. (B–F) RT-qPCR of *MYRACL*, unpaired two-tailed t test (t = 15.11, df = 4); *MBP*, unpaired two-tailed t test (t = 3.243, df = 16); *SOX10*, unpaired two-tailed t test (t = 2.887, df = 16); *OLIG2*, unpaired two-tailed t test (t = 2.182, df = 16); and *PDGFRA*, unpaired two-tailed t test (t = 1.843, df = 16) (n = 9) after OPC transduction with LV-MY versus control (LV-CTR). (G) Representative photomicrographs of IF for neurofilament (NF), MBP, and CASPR in hESC-derived OLs after *MYRACL* overexpression. Maximum intensity projections visualize the increased number of MBP$^+$ myelin segments after *MYRACL* overexpression. Dashed rectangles indicate a selected region, which is presented in higher magnification as single optical section on the top left of the CASPR panel, visualizing the CASPR localization. (H and I) Quantification of total myelin area; unpaired two-tailed t test (t = 6.107, df = 18) and myelin relative to axonal density; unpaired two-tailed t test (t = 6.204, df = 18) following lentiviral treatment (26 sections from n = 10 animals). (J) Quantification of MBP$^+$ internodes with paranodal loops (CASPR$^+$) after *MYRACL* lentiviral overexpression. No significant difference is observed between the numbers of MBP$^+$/CASPR$^+$ and MBP$^+$ internodes within the LV-CTR and the LV-MY groups; however, the total number of internodes is increased after the lentiviral overexpression of *MYRACL*; two-way ANOVA (F(1,56) = 12.57, p = 0.0008). (K and L) Quantification of MBP$^+$ cells; unpaired two-tailed t test (t = 1.132, df = 18) and total axonal area; unpaired two-tailed t test (t = 0.2830, df = 12) following lentiviral transductions (26 sections from n = 10 animals). Scale bar, 10 μm. All bars show means ± SEM and p values given on graphs.

SUPPLEMENTAL INFORMATION

Supplemental information can be found online at https://doi.org/10.1016/j.ymthe.2025.08.011.

REFERENCES

1. Clayton, B.L.L., and Tesar, P.J. (2021). Oligodendrocyte progenitor cell fate and function in development and disease. Curr. Opin. Cell Biol. *73*, 35–40. https://doi.org/10.1016/j.ceb.2021.05.003.
2. Santos, A.K., Vieira, M.S., Vasconcellos, R., Goulart, V.A.M., Kihara, A.H., and Resende, R.R. (2019). Decoding cell signalling and regulation of oligodendrocyte differentiation. Semin. Cell Dev. Biol. *95*, 54–73. https://doi.org/10.1016/j.semcdb.2018.05.020.
3. Li, H., He, Y., Richardson, W.D., and Casaccia, P. (2009). Two-tier transcriptional control of oligodendrocyte differentiation. Curr. Opin. Neurobiol. *19*, 479–485. https://doi.org/10.1016/j.conb.2009.08.004.
4. Mattick, J.S., Amaral, P.P., Carninci, P., Carpenter, S., Chang, H.Y., Chen, L.-L., Chen, R., Dean, C., Dinger, M.E., Fitzgerald, K.A., et al. (2023). Long non-coding RNAs: definitions, functions, challenges and recommendations. Nat. Rev. Mol. Cell Biol. *24*, 430–447. https://doi.org/10.1038/s41580-022-00566-8.
5. Srinivas, T., Mathias, C., Oliveira-Mateos, C., and Guil, S. (2023). Roles of lncRNAs in brain development and pathogenesis: Emerging therapeutic opportunities. Mol. Ther. *31*, 1550–1561. https://doi.org/10.1016/j.ymthe.2023.02.008.
6. Briggs, J.A., Wolvetang, E.J., Mattick, J.S., Rinn, J.L., and Barry, G. (2015). Mechanisms of Long Non-coding RNAs in Mammalian Nervous System Development, Plasticity, Disease, and Evolution. Neuron *88*, 861–877. https://doi.org/10.1016/j.neuron.2015.09.045.
7. Mills, J.D., Kavanagh, T., Kim, W.S., Chen, B.J., Waters, P.D., Halliday, G.M., and Janitz, M. (2015). High expression of long intervening non-coding RNA OLMALINC in the human cortical white matter is associated with regulation of oligodendrocyte maturation. Mol. Brain *8*, 2. https://doi.org/10.1186/s13041-014-0091-9.
8. Simchovitz, A., Hanan, M., Yayon, N., Lee, S., Bennett, E.R., Greenberg, D.S., Kadener, S., and Soreq, H. (2020). A lncRNA survey finds increases in neuroprotective LINC-PINT in Parkinson's disease substantia nigra. Aging Cell *19*, e13115. https://doi.org/10.1111/acel.13115.
9. Zhang, J., Guan, M., Zhou, X., Berry, K., He, X., and Lu, Q.R. (2023). Long Noncoding RNAs in CNS Myelination and Disease. Neuroscientist *29*, 287–301. https://doi.org/10.1177/10738584221083919.
10. Ramos, A.D., Andersen, R.E., Liu, S.J., Nowakowski, T.J., Hong, S.J., Gertz, C., Salinas, R.D., Zarabi, H., Kriegstein, A.R., and Lim, D.A. (2015). The Long Noncoding RNA Pnky Regulates Neuronal Differentiation of Embryonic and Postnatal Neural Stem Cells. Cell Stem Cell *16*, 439–447. https://doi.org/10.1016/j.stem.2015.02.007.
11. Zeng, T., Ni, H., Yu, Y., Zhang, M., Wu, M., Wang, Q., Wang, L., Xu, S., Xu, Z., Xu, C., et al. (2019). BACE1-AS prevents BACE1 mRNA degradation through the sequestration of BACE1-targeting miRNAs. J. Chem. Neuroanat. *98*, 87–96. https://doi.org/10.1016/j.jchemneu.2019.04.001.
12. Vacante, F., Rodor, J., Lalwani, M.K., Mahmoud, A.D., Bennett, M., De Pace, A.L., Miller, E., Van Kuijk, K., de Bruijn, J., Gijbels, M., et al. (2021). CARMN Loss Regulates Smooth Muscle Cells and Accelerates Atherosclerosis in Mice. Circ. Res. *128*, 1258–1275. https://doi.org/10.1161/circresaha.120.318688.
13. Karimi, E., Azari, H., Tahmasebi, A., Nikpoor, A.R., Negahi, A.A., Sanadgol, N., Shekari, M., and Mousavi, P. (2022). LncRNA-miRNA network analysis across the Th17 cell line reveals biomarker potency of lncRNA NEAT1 and KCNQ1OT1 in multiple sclerosis. J. Cell. Mol. Med. *26*, 2351–2362. https://doi.org/10.1111/jcmm.17256.
14. Cao, B., Wang, T., Qu, Q., Kang, T., and Yang, Q. (2018). Long Noncoding RNA SNHG1 Promotes Neuroinflammation in Parkinson's Disease via Regulating miR-7/NLRP3 Pathway. Neuroscience *388*, 118–127. https://doi.org/10.1016/j.neuroscience.2018.07.019.
15. He, D., Wang, J., Lu, Y., Deng, Y., Zhao, C., Xu, L., Chen, Y., Hu, Y.-C., Zhou, W., and Lu, Q.R. (2017). lncRNA Functional Networks in Oligodendrocytes Reveal Stage-Specific Myelination Control by an lncOL1/Suz12 Complex in the CNS. Neuron *93*, 362–378. https://doi.org/10.1016/j.neuron.2016.11.044.
16. Wei, H., Dong, X., You, Y., Hai, B., Duran, R.C.-D., Wu, X., Kharas, N., and Wu, J.Q. (2021). OLIG2 regulates lncRNAs and its own expression during oligodendrocyte lineage formation. BMC Biol. *19*, 132. https://doi.org/10.1186/s12915-021-01057-6.
17. Liang, X., Gong, M., Wang, Z., Wang, J., Guo, W., Cai, A., Yang, Z., Liu, X., Xu, F., Xiong, W., et al. (2024). LncRNA TubAR complexes with TUBB4A and TUBA1A to promote microtubule assembly and maintain myelination. Cell Discov. *10*, 54. https://doi.org/10.1038/s41421-024-00667-y.
18. Zhou, Q., Jiang, Y., Cai, C., Li, W., Leow, M.K.S., Yang, Y., Liu, J., Xu, D., and Sun, L. (2023). Multidimensional conservation analysis decodes the expression of conserved long noncoding RNAs. Life Sci. Alliance *6*, e202302002. https://doi.org/10.26508/lsa.202302002.
19. Dvash, T., Ben-Yosef, D., and Eiges, R. (2006). Human Embryonic Stem Cells as a Powerful Tool for Studying Human Embryogenesis. Pediatr. Res. *60*, 111–117. https://doi.org/10.1203/01.pdr.0000228349.24676.17.
20. Lv, D., Xu, K., Jin, X., Li, J., Shi, Y., Zhang, M., Jin, X., Li, Y., Xu, J., and Li, X. (2020). LncSpA: LncRNA Spatial Atlas of Expression across Normal and Cancer Tissues. Cancer Res. *80*, 2067–2071. https://doi.org/10.1158/0008-5472.Can-19-2687.
21. Mudge, J.M., Ruiz-Orera, J., Prensner, J.R., Brunet, M.A., Calvet, F., Jungreis, I., Gonzalez, J.M., Magrane, M., Martinez, T.F., Schulz, J.F., et al. (2022). Standardized annotation of translated open reading frames. Nat. Biotechnol. *40*, 994–999. https://doi.org/10.1038/s41587-022-01369-0.
22. Li, Y., Zhou, H., Chen, X., Zheng, Y., Kang, Q., Hao, D., Zhang, L., Song, T., Luo, H., Hao, Y., et al. (2021). SmProt: A Reliable Repository with Comprehensive Annotation of Small Proteins Identified from Ribosome Profiling. Genom. Proteom. Bioinform. *19*, 602–610. https://doi.org/10.1016/j.gpb.2021.09.002.
23. Olexiouk, V., Van Criekinge, W., and Menschaert, G. (2018). An update on sORFs.org: a repository of small ORFs identified by ribosome profiling. Nucleic Acids Res. *46*, D497–D502. https://doi.org/10.1093/nar/gkx1130.
24. Spiroski, A.M., Sanders, R., Meloni, M., McCracken, I.R., Thomson, A., Brittan, M., Gray, G.A., and Baker, A.H. (2021). The Influence of the LINC00961/SPAAR Locus Loss on Murine Development, Myocardial Dynamics, and Cardiac Response to Myocardial Infarction. Int. J. Mol. Sci. *22*, 969. https://doi.org/10.3390/ijms22020969.
25. Tian, H., Tang, L., Yang, Z., Xiang, Y., Min, Q., Yin, M., You, H., Xiao, Z., and Shen, J. (2024). Current understanding of functional peptides encoded by lncRNA in cancer. Cancer Cell Int. *24*, 252. https://doi.org/10.1186/s12935-024-03446-7.
26. Liu, H., Zhou, X., Yuan, M., Zhou, S., Huang, Y.E., Hou, F., Song, X., Wang, L., and Jiang, W. (2020). ncEP: A Manually Curated Database for Experimentally Validated ncRNA-encoded Proteins or Peptides. J. Mol. Biol. *432*, 3364–3368. https://doi.org/10.1016/j.jmb.2020.02.022.
27. Tsarouchas, T.M., Zoupi, L., Williams, A., and Gibson, E.M. (2025). Protocol for assessing myelination by human iPSC-derived oligodendrocytes in Shiverer mouse ex vivo brain slice cultures. STAR Protoc. *6*, 103609. https://doi.org/10.1016/j.xpro.2025.103609.
28. Yuen, T.J., Johnson, K.R., Miron, V.E., Zhao, C., Quandt, J., Harrisingh, M.C., Swire, M., Williams, A., McFarland, H.F., Franklin, R.J.M., and Ffrench-Constant, C. (2013). Identification of endothelin 2 as an inflammatory factor that promotes central nervous system remyelination. Brain *136*, 1035–1047. https://doi.org/10.1093/brain/awt024.
29. Dong, X., Chen, K., Cuevas-Diaz Duran, R., You, Y., Sloan, S.A., Zhang, Y., Zong, S., Cao, Q., Barres, B.A., and Wu, J.Q. (2015). Comprehensive Identification of Long Non-coding RNAs in Purified Cell Types from the Brain Reveals Functional LncRNA in OPC Fate Determination. Plos Genet. *11*, e1005669. https://doi.org/10.1371/journal.pgen.1005669.

YMTHE, Volume *33*

Supplemental Information

The lncRNA *MYRACL* regulates human oligodendrocyte maturation and myelination

Themistoklis M. Tsarouchas, Francesca Vacante, Nina-Lydia Kazakou, Laura Wagstaff, Matthew Bennett, Lida Zoupi, Erin M. Gibson, Andrew H. Baker, and Anna Williams

Supplemental Tables

Table S1. qRT-PCR primer efficiency calculated using the "(E = (10^(-1/slope)) – 1) formula.

Primer	Slope	% Efficiency
UBC	-3.282	101.7
MYRACL	-3.2111	104.8
MBP	-3.1677	106.9
SOX10	-3.0131	114.7
OLIG2	-3.0886	110.8
PDGFRα	-3.1583	107.3

Table S2. List of differentially expressed lncRNAs across datasets. (Can be found in the attached file).

Table S3. Correlation analysis between MYRACL and protein-coding genes in human single-nucleus RNA-seq data. The table displays the top positively and negatively correlated protein-coding genes, along with their Ensembl gene IDs, gene symbols, correlation coefficients, and associated p-values.

Ensembl_ID lncRNA	lncRNA_Symbol	Ensembl_ID mRNA Gene	mRNA_Gene_Symbol	Pearson_correlation	Pval
ENSG00000249362	MYRACL	ENSG00000152034	MCHR2	0.9252	0
ENSG00000249362	MYRACL	ENSG00000292336	ASMT	0.8959	0
ENSG00000249362	MYRACL	ENSG00000166569	CPLX4	0.8823	0
ENSG00000249362	MYRACL	ENSG00000220575	HTR5A-AS1	0.8662	0
ENSG00000249362	MYRACL	ENSG00000173786	CNP	0.8201	0
ENSG00000249362	MYRACL	ENSG00000145863	GABRA6	0.8108	0

ENSG00000249362	MYRACL	ENSG00000112706	IMPG1	0.8022	0
ENSG00000249362	MYRACL	ENSG00000265203	RBP3	0.7979	0
ENSG00000249362	MYRACL	ENSG00000197971	MBP	0.7524	0
ENSG00000249362	MYRACL	ENSG00000235718	MFRP	0.7445	0
ENSG00000249362	MYRACL	ENSG00000020129	NCDN	0.7417	0
ENSG00000249362	MYRACL	ENSG00000178394	HTR1A	0.7381	0
ENSG00000249362	MYRACL	ENSG00000203737	GPR52	0.7335	0
ENSG00000249362	MYRACL	ENSG00000109047	RCVRN	0.7212	0
ENSG00000249362	MYRACL	ENSG00000197430	OPALIN	0.7154	0
ENSG00000249362	MYRACL	ENSG00000166159	LRTM2	0.7100	0
ENSG00000249362	MYRACL	ENSG00000184388	PABPC1L2B	0.7092	0
ENSG00000249362	MYRACL	ENSG00000006116	CACNG3	0.7086	0
ENSG00000249362	MYRACL	ENSG00000081148	IMPG2	0.7058	0
ENSG00000249362	MYRACL	ENSG00000168314	MOBP	0.7044	0
ENSG00000249362	MYRACL	ENSG00000125835	SNRPB	-0.9507	8.41E-17
ENSG00000249362	MYRACL	ENSG00000145721	LIX1	-0.9511	7.56E-17
ENSG00000249362	MYRACL	ENSG00000262655	SPON1	-0.9512	7.17E-17
ENSG00000249362	MYRACL	ENSG00000164611	PTTG1	-0.9530	4.17E-17
ENSG00000249362	MYRACL	ENSG00000125170	DOK4	-0.9537	3.38E-17
ENSG00000249362	MYRACL	ENSG00000114698	PLSCR4	-0.9549	2.28E-17
ENSG00000249362	MYRACL	ENSG00000115457	IGFBP2	-0.9560	1.57E-17
ENSG00000249362	MYRACL	ENSG00000125354	SEPTIN6	-0.9562	1.47E-17
ENSG00000249362	MYRACL	ENSG00000165802	NSMF	-0.9565	1.33E-17
ENSG00000249362	MYRACL	ENSG00000127084	FGD3	-0.9616	2.17E-18

Table S4. Summary of coding potential analysis for all transcript isoforms of the lncRNA MYRACL using the CPC2 tool. Each transcript is annotated with its Ensembl Transcript ID, predicted peptide length (if any), Fickett score (reflecting positional nucleotide bias), isoelectric point (pI), ORF integrity (1 = intact ORF, -1 = lacking intact ORF), and predicted coding probability. All transcript isoforms are labeled as noncoding by CPC2, consistent with GENCODE annotations.

Transcript_ID	Peptide length	Fickett_ score	pI	ORF integrity	Coding probability	Label
ENST00000504287.3	57	0.30883	9.60064697	1	0.0258124	noncoding
ENST00000655396.2	82	0.24797	9.6317749	1	0.0585032	noncoding
ENST00000815382.1	20	0.36105	10.3098755	-1	0.134737	noncoding
ENST00000815383.1	19	0.38708	10.3098755	-1	0.10864	noncoding
ENST00000815384.1	20	0.34969	10.3098755	-1	0.147547	noncoding
ENST00000815385.1	20	0.37967	10.3098755	-1	0.115735	noncoding
ENST00000815386.1	20	0.38394	10.3098755	-1	0.111859	noncoding
ENST00000815387.1	20	0.33139	10.3098755	-1	0.168443	noncoding
ENST00000815388.1	20	0.4197	10.3098755	-1	0.0874846	noncoding
ENST00000815389.1	20	0.35712	10.3098755	-1	0.139098	noncoding
ENST00000815390.1	20	0.40561	10.3098755	-1	0.0953708	noncoding
ENST00000815391.1	19	0.33386	10.3098755	-1	0.165638	noncoding
ENST00000815392.1	19	0.41544	8.34283447	1	0.00552503	noncoding
ENST00000815393.1	14	0.45961	10.8344116	1	0.0181856	noncoding
ENST00000815394.1	82	0.2613	9.6317749	1	0.0534975	noncoding
ENST00000815395.1	40	0.25242	5.59637451	1	0.0359882	noncoding
ENST00000815396.1	40	0.27616	5.59637451	1	0.0196133	noncoding
ENST00000815397.1	40	0.30862	5.59637451	1	0.012922	noncoding

ENST00000815398.1	38	0.29826	9.90155029	1	0.0153645	noncoding
ENST00000815399.1	45	0.30763	6.03131104	1	0.0144505	noncoding

Supplemental Methods

Generation of OPCs and Oligodendrocytes from hESCs in-vitro

We obtained ethical permission from the UK Stem Cell Bank for the use of human embryonic stem cells (hESCs). These cells have normal karyotype and have recently undergone single nucleotide polymorphism analysis (SNP) analysis as previously described [1]. Generation of hESC-derived OPCs and OLs was performed as previously described [2]. hESCs were cultured on laminin-521-coated plates (5 μg/ml, Biolamina) using StemMACS™ iPS-Brew XF medium (Miltenyi Biotec) supplemented with 1% Antibiotic Antimycotic Solution (Sigma). To initiate differentiation, hESCs were detached using accutase and resuspended in StemMACS™ iPS-Brew XF medium with 1% Antibiotic Antimycotic Solution and ROCK inhibitor Y-27632 (10 μM, Tocris). Approximately 2×10^6 cells were seeded per well in AggreWell™400 microwell culture plates (Stem Cell Technologies) and incubated overnight to form embryoid bodies. Next, the embryoid bodies were transferred to a rotary shaker, and cultured in a chemically defined neuralization medium containing a 1:1 mix of F12 and Iscove's modified Dulbecco's medium (Invitrogen), with chemically defined Lipid concentrate 100 (Invitrogen), BSA (5 mg/ml, Sigma), monothioglycerol (450 μM, Sigma), insulin (7 mg/ml, Roche), transferrin (15 mg/ml, Roche), and 1% Antibiotic Antimycotic Solution. Activin inhibitor SB 431542 (10 μM, Sigma), N-acetyl cysteine (1 mM, Sigma), and LDN193189 (0.1 μM, Stratech) were added for neural induction. After 10 days, the neural spheres were caudalized for an additional 7 days in chemically defined medium

supplemented with N-acetyl cysteine (1 mM, Sigma), heparin (5 μg/ml, Sigma), retinoic acid (0.1 μM, Sigma), and basic fibroblast growth factor (FGF-2; 10 ng/ml, PeproTech). Neural conversion was assessed morphologically when cells were plated on laminin-coated plates (10 μg/ml, Sigma). Following neural induction, the spheres were cultured in advanced DMEM medium containing 0.5% GlutaMAX (Invitrogen), 1% N2 (Invitrogen), 1% B27™ (Invitrogen), 5 μg/ml heparin (Sigma), and 1% Antibiotic Antimycotic Solution. For ventralization, the medium was supplemented with purmorphamine (1 μM, Calbiochem), retinoic acid (1 μM, Sigma), and FGF-2 (10 ng/ml, PeproTech) for 7 days, after which FGF-2 was withdrawn for 14 days. Next, hOPC proliferation was enhanced by adding FGF-2 (10 ng/ml), alongside T3 (60 ng/ml, Sigma), PDGFα (20 ng/ml, PeproTech), SAG (1 μM, Calbiochem), purmorphamine (1 μM, Sigma), and IGF-1 (10 ng/ml, PeproTech). After 2 weeks, the spheres were dissociated using the Worthington papain dissociation system according to the manufacturer's protocol. Cells were plated at a density of 4×10^4 cells in 40 μl droplets on coverslips coated with poly-ornithine (1:100, Sigma), laminin (10 μg/ml, Sigma), fibronectin (20 μg/ml, Sigma), and Matrigel (Corning), whereas 1×10^6 cells per well were used for coated 6-well plates. For final differentiation into oligodendrocytes, cells were maintained for 1 week in advanced DMEM supplemented with 0.5% GlutaMAX, 1% N2, 1% B27, heparin (5 μg/ml), and 1% Antibiotic Antimycotic Solution, along with IGF-1 (10 μg/ml, PeproTech), T3 (60 μg/ml, Sigma), and ITS (Insulin-Transferrin-Sodium Selenite; 1:100, Sigma).

Immunofluorescence

Coverslips were rinsed with PBS and fixed with 4% PFA for 10 minutes. After fixation, coverslips were washed with PBS and blocked in a solution of PBS containing 10% horse serum (v/v) and 0.1% Triton-X (v/v) for 1 hour at room temperature. Primary antibodies anti-MBP (rat monoclonal, MCA409S, BioRad, 1:250), anti-Olig2 (goat polyclonal, AF2418, R&D systems, 1:400), anti-O4 (mouse monoclonal, MAB1326, R&D systems, 1:1000), anti-PDGFRα (rabbit monoclonal, 3174, Cell signalling, 1:200) were diluted in the blocking solution and incubated overnight at 4°C. The next day, coverslips were washed with PBS and incubated with Alexa-conjugated secondary antibodies (1:1000) and DAPI (1:1000) for nuclear staining, in PBS, for 2 hours at room temperature. Following incubation, coverslips were washed again with PBS and mounted using Fluoromount.

Assessing potential for micropeptide production

To evaluate the coding potential of the MYRACL (LINC02488), we first referenced the GENCODE v44 annotation used throughout our snRNA-seq analyses, which classifies this gene as non-coding based on curated experimental and computational evidence. To further validate this, we ran the CPC2 (Coding Potential Calculator 2)[3] on all MYRACL transcripts listed in Ensembl v114. CPC2 evaluates open reading frame structure and composition using four predictive features. The Fickett score measures the positional bias of nucleotides within the RNA sequence, which can indicate whether the sequence is likely to be coding. ORF length is also considered, as protein-coding transcripts typically contain longer and higher-quality open reading frames than non-coding RNAs. ORF integrity assesses whether the identified ORF is structurally valid and complete, further supporting its potential to encode a protein. Lastly, the

isoelectric point (pI) of hypothetical peptides is calculated, based on the premise that peptides derived from non-coding sequences often exhibit different biochemical properties, such as pI, compared to genuine protein-coding sequences. None of the MYRACL transcripts displayed a coding potential score indicative of translation, and no open reading frame greater than 100 amino acids was detected. To explore potential translation of small peptides (sORFs <100 AA), we queried the GWIPS-viz Ribo-seq browser[4], which aggregates ribosome profiling data from 1792 public experiments. No ribosomal coverage indicative of active translation was found at the MYRACL locus. In addition, we manually cross-checked two recently published brain-specific sORF annotations[5,6] and found no annotated translated ORFs overlapping MYRACL. These analyses support the classification of MYRACL as a bona fide lncRNA with no detectable evidence of coding activity across available datasets.

Re-analysis of published single nucleus RNA sequencing datasets

Publicly available single-nucleus RNA sequencing (snRNA-seq) datasets from Jäkel et al.2019[7], the GTEx Consortium[8], Macnair et al.2024[9] were downloaded from the Gene Expression Omnibus (GEO) under accession number GSE118257, the GTex portal, and the European Genome-Phenome Archive (EGA) as dataset EGA: EGAD0000100916 respectively, and the information was correlated with the LncRNA Spatial Atlas of Expression (LncSpA)[10]. Raw FASTQ files were processed using Cell Ranger (v7.2.0) with reference transcriptome incorporating protein-coding and long non-coding RNAs. The reference genome was constructed using cellranger mkref with GENCODE v44, ensuring inclusion of all annotated lncRNA genes. Expression matrices were generated using cellranger count and used as input for downstream analysis. Filtered gene-barcode matrices were imported into Python and processed

using Scanpy (v1.9.6). Cells were filtered based on standard quality control metrics (cells with fewer than 200 detected genes, fewer than 500 total UMI counts, or more than 5% mitochondrial gene expression were excluded from further analysis. Genes detected in fewer than 3 cells were also removed. Gene expression counts were normalized to a total of 10,000 counts per cell. To integrate data across samples and conditions, we employed Seurat v4's reciprocal PCA (RPCA) framework developed by the Satija lab[11]. This method aligns datasets into a shared transcriptional space by identifying anchors across batches while correcting for technical variability. Following integration, we assigned cell type identities based on canonical marker gene expression for oligodendrocyte precursor cells (PDGFRA, CSPG4) and mature oligodendrocytes (MBP, MOG, PLP1). Differential gene expression analysis between annotated OPC and oligodendrocyte clusters was performed using a pseudobulk approach. Specifically, counts from nuclei of the same cell type within each sample were aggregated and used as input for DESeq2. This strategy enables robust detection of biologically meaningful differences while controlling for donor-specific variation. Results were filtered to retain only genes annotated as lncRNAs in GENCODE v44. lncRNAs with log2 fold change ≥ 0.5 and adjusted p-value < 0.05 (Benjamini-Hochberg FDR correction) were considered significantly differentially expressed.

Co-expression analysis

To explore the potential regulatory role of MYRACL in oligodendrocyte lineage development, we performed a targeted co-expression analysis. The expression levels of MYRACL were correlated with all protein-coding genes in mature oligodendrocytes

according to Pearson correlation. Genes with a correlation coefficient $|r| \geq 0.7$ and adjusted $p < 0.05$ were considered significantly co-expressed.

qRT-PCR

RNA was extracted using the RNeasy Mini Kit (Qiagen) following the manufacturer's protocol with minor adjustments. For each sample, 1×10^6 oligodendroglia cells were disrupted in Buffer RLT, mixed with ethanol, and processed through RNeasy spin columns with successive washing steps using Buffers RW1 and RPE. RNA was eluted in RNase-free water and assessed for quality and quantity using a NanoDrop™ Lite spectrophotometer before storage at -80°C. Complementary DNA (cDNA) was synthesized using the iScript™ cDNA synthesis kit (Bio-Rad), with 500 ng RNA reverse-transcribed in a thermocycler under specified conditions indicated by the kit's manufacturer. Quantitative PCR (qPCR) was performed using SsoAdvanced™ Universal SYBR® Green Supermix (Bio-Rad) with UBC as housekeeping gene. Reaction volumes were prepared with primers, cDNA, and nuclease-free water to a total of 10 µl per reaction. Primers were reconstituted into 100 µM stock concentration. The stock was then diluted into 10 µM, and for the qRT-PCR, 1 µL of the 10 µM primer solution was used for a 10 µL reaction volume. Primer efficiencies were determined from standard curves generated by serial cDNA dilutions and calculated using the slope-based equation: $E = 10^{(-1/slope)}$ (**Table S1**). Relative gene expression was analysed using the Pfaffl method, which incorporates individual amplification efficiencies and Ct values to generate accurate expression ratios[12]. The primers used are: UBC: F-TTGAGCCCAGTGACACCATC, R-TTGTAGTCAGACAGGGTGCG, MBP: F-AGCGCACCTGTGATTGATAG, R- AAGACGCGTTTTGGCATCAC, SOX10: F-CCTCACAGATCGCCTACACC, R-CATATAGGAGAAGGCCGAGTAGA, MYRACL:

F-AGTTCAGTTTTATTGGTTGCACGC, R-CTGGTTAGTTTGTAGGGCCTT, OLIG2: F-ATGCACGACCTCAACATCGCCA, R-ACCAGTCGCTTCATCTCCTCCA, PDGFRα: F-GACTTTCGCCAAAGTGGAGGAG, R-AGCCACCGTGAGTTCAGAACGC.

GapmeR mediated knockdown of MYRACL

To perform the transfection and GapmeR knockdown, hESC-derived OPCs are first plated on Day 1 in proliferation medium at a density of 2 × 10^6 cells per well in a 6-well plate or 4 x 10^4 on coverslips. On Day 2, transfection complexes are prepared by diluting 5 µL Lipofectamine-RNAiMAX (ThermoFisher) in Opti-MEM medium (final volume of 500 µL per well for coverslips, or 1 mL per well for 6-well plates) and incubating the mixture at room temperature for 5 minutes. 40 nM of GapmeR was used for the experiments. Equal volumes of the diluted RNAiMAX and GapmeR are then combined (e.g., 500 µL RNAiMAX + 500 µL GapmeR), gently mixed, and incubated at room temperature for 20 minutes to allow transfection complexes to form. During this time, the cells are prepared by removing the growth medium and washing each well with Opti-MEM. Subsequently, the transfection mixture is added to each well, ensuring no pipetting up and down to avoid liposome disruption, and the cells are incubated at 37°C for 6 hours. Following this incubation, one volume of OPC proliferation medium is added to each well, and the cells are returned to 37°C incubation until the next day. On the morning of Day 3, the transfection medium is replaced with one volume of OPC proliferation medium, and the cells are maintained in this condition for 48 hours. On Day 5, the samples are harvested, RNA is extracted, and knockdown efficiency is assessed by qPCR. The following GapmeRs were designed and generated by QIAGEN to target MYRACL (Gap-MY) (Cat. No. LG00790906-GCGTGCAACCAATAAA, and Cat. No. LG00790907-GAAGAATGGCAAACGC).

Lentiviral overexpression of MYRACL transcripts

In order to overexpress MYRACL within the hESC-derived oligodendroglia, 3rd generation lentiviruses including MYRACL exonic sequence, (Ensembl annotation GRCh38.p13) was generated by the Biomolecular Core Facility of the University of Edinburgh, UK. To perform the transduction, hESC-derived OPCs are first plated on Day 1 in proliferation medium at a density of 2×10^6 cells per well in a 6-well plate or 4×10^4 on coverslips. On Day 2, infection complexes are prepared by diluting 0.5 µL Polybrene (ThermoFisher) in Opti-MEM medium (final volume of 500 µL per well for coverslips, or 1 mL per well for 6-well plates). The generated lentiviruses were used at 40 MOI and were diluted into the polybrene mix and then were added to the cells. The cells are incubated at 37°C overnight. Following this incubation, one volume of OPC proliferation medium is added to each well, and the cells are returned to 37°C incubation until the next day. On the morning of Day 3, the transfection medium is replaced with one volume of OPC proliferation medium, and the cells are maintained in this condition for 48 hours. On Day 5, the samples are harvested, RNA is extracted, and overexpression efficiency is assessed by qPCR.

Myelination assay on organotypic Shiverer brain sections ex vivo

The generation of organotypic Shiverer brain sections and the myelination assay was performed as previously described[13]. Briefly, P0–P2 Shiverer pups were euthanized following the standard UK Home Office regulations under project licences PADF15B79 and PP1335335, and their brains were extracted and placed in cold Hibernate™-A medium (Thermo Scientific) on ice. The brains were mounted on a vibratome (LEICA) and coronal cortical slices (250–300 µm) were prepared in cold Hibernate™-A medium and transferred to Millicell cell culture inserts (Merck-Millipore). The slices were

cultured in a warm medium containing 50% MEM (Life Technologies), 25% Earle's Balanced Salt Solution (Life Technologies), 25% heat-inactivated horse serum (Thermo Scientific), 1% Glutamax™ supplement (Thermo Scientific), 1% penicillin–streptomycin, 0.5% Amphotericin B (Thermo Scientific), and 6.5 mg/ml glucose (Sigma-Aldrich).

Slices were maintained in serum-containing medium and then transitioned to serum-free medium containing DMEM/F12 (Thermo Scientific), 1% B-27™ supplement, 0.5% N2 supplement, 1% Glutamax™ supplement, 1% penicillin–streptomycin, and 0.5% Amphotericin B. All cultures were incubated at 37°C and 5% CO2, with medium changes every two days. After 7 days in culture, 100,000 hESC-derived OPCs cells were seeded onto each cortical slice and co-cultured for an additional four weeks. Then, slices were washed once with 1x PBS before being fixed in 4% paraformaldehyde (PFA) for 1 hour at room temperature. After fixation, the slices were rinsed in 1x PBS and blocked for 2 hours at room temperature in a solution containing 3% heat-inactivated horse serum, 2% BSA (Sigma-Aldrich, A7906), and 0.5% Triton X-100 in 1x PBS. Following the blocking step, the slices were incubated at 4°C for 48 hours with primary antibodies (anti-MBP, rat monoclonal, MCA409S, BioRad, 1:250), anti-Neurofilament-H (chicken polyclonal, 822601, BioLegend, 1:100), anti-Caspr (rabbit polyclonal, ab34151, Abcam, 1:1000) diluted in the blocking solution. After primary antibody incubation, slices were washed three times with blocking solution and then incubated overnight at 4°C with the appropriate secondary antibodies. Finally, the slices were washed in 1x PBS, counterstained with Hoechst 33342 solution, and mounted onto glass microscope slides using Fluoromount.

Subcellular cellular fractionation

Isolation of the nuclear and cytoplasmic fragments of hESC-derived OPCs and oligodendrocytes was performed using the PARIS™ Kit (Invitrogen, AM1921) according to manufacturer's instructions. For the cellular fractionation experiments, triplicates of $5*10^5$ hESCs, $6*10^6$ OPCs and $6*10^6$ Oligodendrocytes were used.

RNAscope

To localize expression of MYRACL within the human oligodendroglia, RNAscope was performed in human ESC-derived oligodendroglia cultures using the RNAscope™ Multiplex Fluorescent Reagent Kit v2 (ACD), according to manufacturer's instructions using a customized probes specifically binding to MYRACL and OPALIN transcripts (RNAscope™ Probe- Hs-OPALIN, Cat No. 579861, RNAscope™ Probe- Hs-MYRACL-C2, Cat No. 1585881-C2, RNAscope™ Probe- Hs-MYRACL-C1, Cat No. 1585871-C1).

Statistical analysis

The experimenter was blinded to the experimental conditions when performing imaging and analysis of data. Data were checked for normality and parametric tests were used for statistical analysis. One-way ANOVA followed by Bonferroni multiple comparisons correction or Student's t-Test were performed using GraphPad Prism 10. Data presented as means +/- SEM with data points of individual biological replicates. Illustrations created with BioRender.com.

Supplemental references

1. Wagstaff, L.J., Bestard-Cuche, N., Kaczmarek, M., Fidanza, A., McNeil, L., Franklin, R.J.M., and Williams, A.C. (2024). CRISPR-edited human ES-derived oligodendrocyte progenitor cells improve remyelination in rodents. Nature Communications *15*, 8570. 10.1038/s41467-024-52444-w.
2. Livesey, M.R., Magnani, D., Cleary, E.M., Vasistha, N.A., James, O.T., Selvaraj, B.T., Burr, K., Story, D., Shaw, C.E., Kind, P.C., et al. (2016). Maturation and electrophysiological properties of human pluripotent stem cell-derived oligodendrocytes. Stem Cells *34*, 1040-1053. 10.1002/stem.2273.
3. Kang, Y.J., Yang, D.C., Kong, L., Hou, M., Meng, Y.Q., Wei, L., and Gao, G. (2017). CPC2: a fast and accurate coding potential calculator based on sequence intrinsic features. Nucleic Acids Res *45*, W12-w16. 10.1093/nar/gkx428.
4. Michel, A.M., Fox, G., M. Kiran, A., De Bo, C., O'Connor, P.B.F., Heaphy, S.M., Mullan, J.P.A., Donohue, C.A., Higgins, D.G., and Baranov, P.V. (2013). GWIPS-viz: development of a ribo-seq genome browser. Nucleic Acids Research *42*, D859-D864. 10.1093/nar/gkt1035.
5. Duffy, E.E., Finander, B., Choi, G., Carter, A.C., Pritisanac, I., Alam, A., Luria, V., Karger, A., Phu, W., Sherman, M.A., et al. (2022). Developmental dynamics of RNA translation in the human brain. Nat Neurosci *25*, 1353-1365. 10.1038/s41593-022-01164-9.
6. Chothani, S.P., Adami, E., Widjaja, A.A., Langley, S.R., Viswanathan, S., Pua, C.J., Zhihao, N.T., Harmston, N., D'Agostino, G., Whiffin, N., et al. (2022). A high-resolution map of human RNA translation. Molecular Cell *82*, 2885-2899.e2888. https://doi.org/10.1016/j.molcel.2022.06.023.
7. Jäkel, S., Agirre, E., Mendanha Falcão, A., van Bruggen, D., Lee, K.W., Knuesel, I., Malhotra, D., ffrench-Constant, C., Williams, A., and Castelo-Branco, G. (2019). Altered human oligodendrocyte heterogeneity in multiple sclerosis. Nature *566*, 543-547. 10.1038/s41586-019-0903-2.
8. Consortium, T.G., Aguet, F., Anand, S., Ardlie, K.G., Gabriel, S., Getz, G.A., Graubert, A., Hadley, K., Handsaker, R.E., Huang, K.H., et al. (2020). The GTEx Consortium atlas of genetic regulatory effects across human tissues. Science *369*, 1318-1330. doi:10.1126/science.aaz1776.
9. Macnair, W., Calini, D., Agirre, E., Bryois, J., Jäkel, S., Smith, R.S., Kukanja, P., Stokar-Regenscheit, N., Ott, V., Foo, L.C., et al. (2025). snRNA-seq stratifies multiple sclerosis patients into distinct white matter glial responses. Neuron *113*, 396-410.e399. 10.1016/j.neuron.2024.11.016.
10. Lv, D., Xu, K., Jin, X., Li, J., Shi, Y., Zhang, M., Jin, X., Li, Y., Xu, J., and Li, X. (2020). LncSpA: LncRNA Spatial Atlas of Expression across Normal and Cancer Tissues. Cancer Res *80*, 2067-2071. 10.1158/0008-5472.Can-19-2687.
11. Hao, Y., Stuart, T., Kowalski, M.H., Choudhary, S., Hoffman, P., Hartman, A., Srivastava, A., Molla, G., Madad, S., Fernandez-Granda, C., and Satija, R. (2024). Dictionary learning for integrative, multimodal and scalable single-cell analysis. Nature Biotechnology *42*, 293-304. 10.1038/s41587-023-01767-y.
12. Pfaffl, M.W. (2001). A new mathematical model for relative quantification in real-time RT-PCR. Nucleic Acids Res *29*, e45. 10.1093/nar/29.9.e45.

13. Tsarouchas, T.M., Zoupi, L., Williams, A., and Gibson, E.M. (2025). Protocol for assessing myelination by human iPSC-derived oligodendrocytes in Shiverer mouse ex vivo brain slice cultures. STAR Protoc *6*, 103609. 10.1016/j.xpro.2025.103609.
